# Supplementary material for: Spatial expression analyses of the putative oncogene ciRS-7 in cancer reshape the microRNA sponge theory
Source: Nat Commun. 2020 Sep 11;11:4551. doi: 10.1038/s41467-020-18355-2 (PMC7486402; doi:10.1038/s41467-020-18355-2)
Supplement: Supplementary file 1 — Supplementary Information [file 41467_2020_18355_MOESM1_ESM.pdf]

Spatial expression analyses of the putative oncogene ciRS-7 in cancer reshape the microRNA sponge theory

Kristensen *et al*.

Supplementary Tables

Supplementary Table 1. Primers and probes used in the study.

Supplementary Table 2. Target sequences for the NanoString nCounter probes.

Supplementary Figures

Supplementary Figure 1. RNA chromogenic in situ hybridization (CISH) for ciRS-7 in colon adenocarcinoma.

Supplementary Figure 2. RNA chromogenic in situ hybridization (CISH) for ciRS-7 in colon adenocarcinoma using TMAs.

Supplementary Figure 3. RNA chromogenic in situ hybridization (CISH) for ciRS-7 in HEK293T ciRS-7 knock-out and wild-type cells.

Supplementary Figure 4. FOS immunohistochemistry in colon adenocarcinoma.

Supplementary Figure 5. Dual color FOS reporter assay in HEK293T cells.

Supplementary Figure 6. miR-7 chromogenic in situ hybridization (CISH) in colon adenocarcinoma and control tissues.

Supplementary Figure 7. Correlations between miR-7 target genes and circRNAs that do not contain miR-7 binding sites.

Supplementary Figure 8. RNA chromogenic in situ hybridization (CISH) for ciRS-7 in various malignant tumors.

Supplementary Table 1. Primers and probes used in the study.

Primers for cloning

	Forward	Reverse
ciRS-7 sgRNA 1	CACCGCAATGTCCAGGGTTTCCGA	CAAATCGGAAACCCTGGACATTGC
ciRS-7 sgRNA 2	CACCGCTCTCACAACAGGCTAGCC	CAAAGGCTAGCCTGTTGTGAGAGC
ppC *FOS* 3'UTR	GATTGCGGCCGCAAGGGGAGGCAGCCGGCA	GATTTCTAGATTGGTCGCATTCAACTTAAA TGCT
ppC 6x miR-7	GTAGTGATTTTGTTGTGTCTTCCAGTCGAG	CCTCGACTGGAAGACACAACAAAATCACTA
psiCheck2 *FOS* 3'UTR	GATTCTCGAGAAGGGGAGGCAGCCGGCA	GATTACTAGTTTGGTCGCATTCAACTTAAA TGCT
psiCheck2 3x miR-7	TCGAAAGGCTTCATGTCTTCCATACATTATGT GTCTTCCAGACCAGATTAGTCTTCCA	TGGAAGACTAATCTGGTCTGGAAGACACA TAATGTATGGAAGACATGAAGCCTT

Primers for genomic DNA PCR

	Forward	Reverse
ciRS-7 genomic	CCAATATCCAGGGCTTCTCA	TGGTTGGGGAATAAAAGCTG

Primers for RT-qPCR

	Forward	Reverse
ciRS-7	ACGTCTCCAGTGTGCTGA	CTTGACACAGGTGCCATC
FOS	CCGGGGATAGCCTCTCTTAC	GTGGGAATGAAGTTGGCACT
GAPDH	GTCAGCCGCATCTTCTTTTG	GCGCCCAATACGACCAAATC

Probes for northern blot

miR-7	ACAACAAAATCACTAGTCTTCCA
miR-15b	TGTAAACCATGATGTGCTGCTA
U6	ATCTCCAGGTCTTCCAGCATC

Supplementary Table 2. Target sequences for the NanoString nCounter probes.

CLASS	NAME	SEQUENCE (sense strand 5'->3')
circRNA	CDR1as (ciRS-7)	AACGTCTCCAGTGTGCTGATCTTCTGACATTCAGGTCTTCCAGTGTCTGCAATATCCAGGGTTTCCGATGGCACCTGTGTCAAGGTCTTCCAACAACTCC
circRNA	circSMARCA5	AGTCAAGTGTTTATAACTTCGAAGGAGAAGACTATAGAGAAAAACAAAAGGGAGGCTTGTGGATCAGAATCTGAACAAAATTGGGAAAGATGAAATGCTT
circRNA	circCDYL	CGGCCCTGTGACTGCAGCCATGGCCACAGGCTTAGCTGTTAACGGGAAAGGTTGAAAGGATTGTTGACAAAAGGAAAAATAAAAAAGGGAAGACAGAGTA
circRNA	circZNF609	AGGAAGGGGAGAATGAGTGTCGCCTGCTAAAGAAAGTCAAGTCTGAAAAGCAATGATGTTGTCCACTGGGCATGTACTGACCAATGTGGCAGGTCTGAGA
circRNA	circFBXW7	ACCTGCCCGTTCACCAACTCTCCTCCCCATTCTATACAAAAACAACAAAAGATTACTTCCTTAGGATAGATTGCCAGAAGTGGAGTTACTGGGTCAGAGG
circRNA	circSLC8A1	GGATTTTGAGGACACTTGTGGAGAGCTCGAATTCCAGAATGATGAAATTGTTAGGTTGTGACAGTTGGAAGTGTCATGTACAACATGCGGCGATTAAGTC
circRNA	circHIPK3	TGTATCAAAGACTGTTTGTTCAACATATCTACAATCTCGGTACTACAGGTATGGCCTCACAAGTCTTGGTCTACCCACCATATGTTTATCAAACTCAGTC
circRNA	circZNF91	AAGCATTACAAATATGAAGAGGCATTATTTATGACCTTTTCTATGGAAAGGTATATGTCCTCATTTTCCTCAAGACTTTTGGCCAGAGCAGAGCATGGAA
circRNA	circZKSCAN1	GTCCCACTTCAAACATTCGTCTCGGAAACCCCGCCTCTTACAGTCACGAGGAATAGTAAAGAAACACATCATAAAACCTCCCAGGACATAAAGGTGAGCA
circRNA	circCCDC66	CTCAGATTGAGGAACGAGACAGACGACGACAAAAACAATTAGAGCATCAGGAAACAGTACTGCTGGAGCACCCTTTCAGTGCTGTGAAACAAGAACTGCA
circRNA	circFAT3	CATCAGGGATGGCAGTGGTCTTGGAAGGTTCAGTATAGACGACGAGAGTGGATGGAAGTATGATGTGATGGATATAATTATGGGACACTGTGTGGGCACA
circRNA	circRP11-255H23.2	AGAAGTGTGAAGAATGTGACAAAGCCTTTAAATGATTGTCACACTTGATTGTAGGTATATGCCCTCATTTTGCTTAAGACCTCTGGCCAGAGCAGGGCAT
lncRNA	LINC00632_T1	GATCAAATGGAGGAGATGGAAGATTGAGAGCAGAGTGCAAGAATGGGAAGAGGGCTTCGTGCCCTGTGCACGTTTGCCACCTAGTGACCAAACTGAATGG
lncRNA	LINC00632_T2	TAGTTACCAGCAAGGCCTCTAGACCCATACTAACTGGGTTTGAATCAAGGTCCCGTCCCTTGCTGATTGTATCACCTTGGGAGGAGATGGAAGATTGAGA
lncRNA	LINC00632_T3	TGCAAAGACGAGACGAGTCCTCCCCAGAGAGGGGAGGCGGTTAAGGAGAGGAGATTGCGTGGCTCGGAGCAGCCAGACCCGATGGTGTGCTTCTCACCTC
miR-7 target gene	*EGFR*	ACATCCTGCCGGTGGCATTTAGGGGTGACTCCTTCACACATACTCCTCCTCTGGATCCACAGGAACTGGATATTCTGAAAACCGTAAAGGAAATCACAGG
miR-7 target gene	*RAF1*	AAAGATCCTAAAGGTTGTCGACCCAACCCCAGAGCAATTCCAGGCCTTCAGGAATGAGGTGGCTGTTCTGCGCAAAACACGGCATGTGAACATTCTGCTT
miR-7 target gene	*PIK3CD*	GAATCAACCGCGAGCGTGTCCCATTCATCCTCACCTACGACTTTGTCCATGTGATTCAGCAGGGGAAGACTAATAATAGTGAGAAATTTGAACGGTTCCG
miR-7 target gene	*PAK1*	TGAACCACTTCCTGTCACTCCAACTCGGGACGTGGCTACATCTCCCATTTCACCTACTGAAAATAACACCACTCCACCAGATGCTTTGACCCGGAATACT
miR-7 target gene	*IRS1*	TTGATGGTGGCATCAAACTACCGATTTAAAACTGGAAGTTGCTGGTACTCAAACCAAAAGTTCATACTCTGGCGACACGAAGGGTTTCCTTTGAGCAACG
miR-7 target gene	*IRS2*	GCGCCGAAACGGGTGATCGCTCTCGACTGCTGCCTGAACATCAACAAGCGCGCCGACGCCAAGCACAAGTACCTGATCGCCCTCTACACCAAGGACGAGT
miR-7 target gene	*MTOR*	TCACTCTTGCCCTCCGAACGCTTGGCAGCTTTGAATTTGAAGGCCACTCTCTGACCCAATTTGTTCGCCACTGTGCGGATCATTTCCTGAACAGTGAGCA
miR-7 target gene	*CDK1*	GGTACCTATGGAGTTGTGTATAAGGGTAGACACAAAACTACAGGTCAAGTGGTAGCCATGAAAAAAATCAGACTAGAAAGTGAAGAGGAAGGGGTTCCTA
miR-7 target gene	*EIF4E*	TGGAGAATCTTTTGATGACTACAGTGATGATGTATGTGGCGCTGTTGTTAATGTTAGAGCTAAAGGTGATAAGATAGCAATATGGACTACTGAATGTGAA
miR-7 target gene	*KLF4*	CGAGCATTTTCCAGGTCGGACCACCTCGCCTTACACATGAAGAGGCATTTTTAAATCCCAGACAGTGGATATGACCCACACTGCCAGAAGAGAATTCAGT
miR-7 target gene	*IGF1-R*	TCTCCTGAGTCCCTCAAGGATGGAGTCTTCACCACTTACTCGGACGTCTGGTCCTTCGGGGTCGTCCTCTGGGAGATCGCCACACTGGCCGAGCAGCCCT
miR-7 target gene	*RB1*	CCTATCTCCGGCTAAATACACTTTGTGAACGCCTTCTGTCTGAGCACCCAGAATTAGAACATATCATCTGGACCCTTTTCCAGCACACCCTGCAGAATGA
miR-7 target gene	*SP1*	AGCCCTGGTGCTACTTGCTTGAAGTTTTCAGTGTAAGTACCCTGATGCCTTTTGGACCTTGGGATCAGATCAAGAGTTTTGGAGATCAGGTACCAAGGAA
miR-7 target gene	*HOXB13*	CCACCAGGGTTCCCAAAGAACCTGGCCCAGTCATAATCATTCATCCTGACAGTGGCAATAATCACGATAACCAGTACTAGCTGCCATGATCGTTAGCCTC
miR-7 target gene	*CCNE1*	CCTCCAGACACCAGTGCGTGCTCCCGATGCTGCTATGGAAGGTGCTACTTGACCTAAGGGACTCCCACAACAACAAAAGCTTGAAGCTGTGGAGGGCCAC
miR-7 target gene	*FAK*	TAGGAGCACGTCTTGCTACCCTCTTTTGAAGATGTTCTCTAGCCTTCCACCAGCAGCGAGGAATTAACCCTGTGTCCTCAGTCGCCAGCACTTACAGCTC
miR-7 target gene	*YY1*	GACCCTGGAGGGCGAGTTCTCGGTCACCATGTGGTCCTCAGATGAAAAAAAAGATATTGACCATGAGACAGTGGTTGAAGAACAGATCATTGGAGAGAAC
miR-7 target gene	*NR4A3*	GTCGTCTGCCTTCCAAACCAAAGAGCCCATTACAACAGGAACCTTCTCAGCCCTCTCCACCTTCTCCTCCAATCTGCATGATGAATGCCCTTGTCCGAGC
miR-7 target gene	*ACK1*	GCCATGGACGACTTCATCCGGGAGGTCAATGCCATGCACTCGCTCGACCACCGAAACCTCATCCGCCTCTACGGGGTGGTGCTCACGCCGCCCATGAAGA
miR-7 target gene	*FOS*	ACTCAAGTCCTTACCTCTTCCGGAGATGTAGCAAAACGCATGGAGTGTGTATTGTTCCCAGTGACACTTCAGAGAGCTGGTAGTTAGTAGCATGTTGAGC
Reference gene	*GAPDH*	GGTCTCCTCTGACTTCAACAGCGACACCCACTCCTCCACCTTTGACGCTGGGGCTGGCATTGCCCTCAACGACCACTTTGTCAAGCTCATTTCCTGGTAT
Reference gene	*ACTB*	TGCAGAAGGAGATCACTGCCCTGGCACCCAGCACAATGAAGATCAAGATCATTGCTCCTCCTGAGCGCAAGTACTCCGTGTGGATCGGCGGCTCCATCCT
Reference gene	*PUM1*	CTGGGGAACATCAGATCATTCAGTTTCCCAGCCAATCATGGTGCAGAGAAGACCTGGTCAGAGTTTCCATGTGAACAGTGAGGTCAATTCTGTACTGTCC
Reference gene	*SF3A1*	CTTCTAAGCCAGTTGTGGGGATTATTTACCCTCCTCCAGAGGTCAGAAATATTGTTGACAAGACTGCCAGCTTTGTGGCCAGAAACGGGCCTGAATTTGA
Reference gene	*B2M*	GTCTGGGTTTCATCCATCCGACATTGAAGTTGACTTACTGAAGAATGGAGAGAGAATTGAAAAAGTGGAGCATTCAGACTTGTCTTTCAGCAAGGACTGG
Reference gene	*HPRT*	TGTGATGAAGGAGATGGGAGGCCATCACATTGTAGCCCTCTGTGTGCTCAAGGGGGGCTATAAATTCTTTGCTGACCTGCTGGATTACATCAAAGCACTG
Reference gene	*MRPL19*	GGAAGTATTCTTCGTGTTACTACAGCTGACCCATATGCCAGTGGAAAAATCAGCCAGTTTCTGGGGATTTGCATTCAGAGATCAGGAAGAGGACTTGGAG

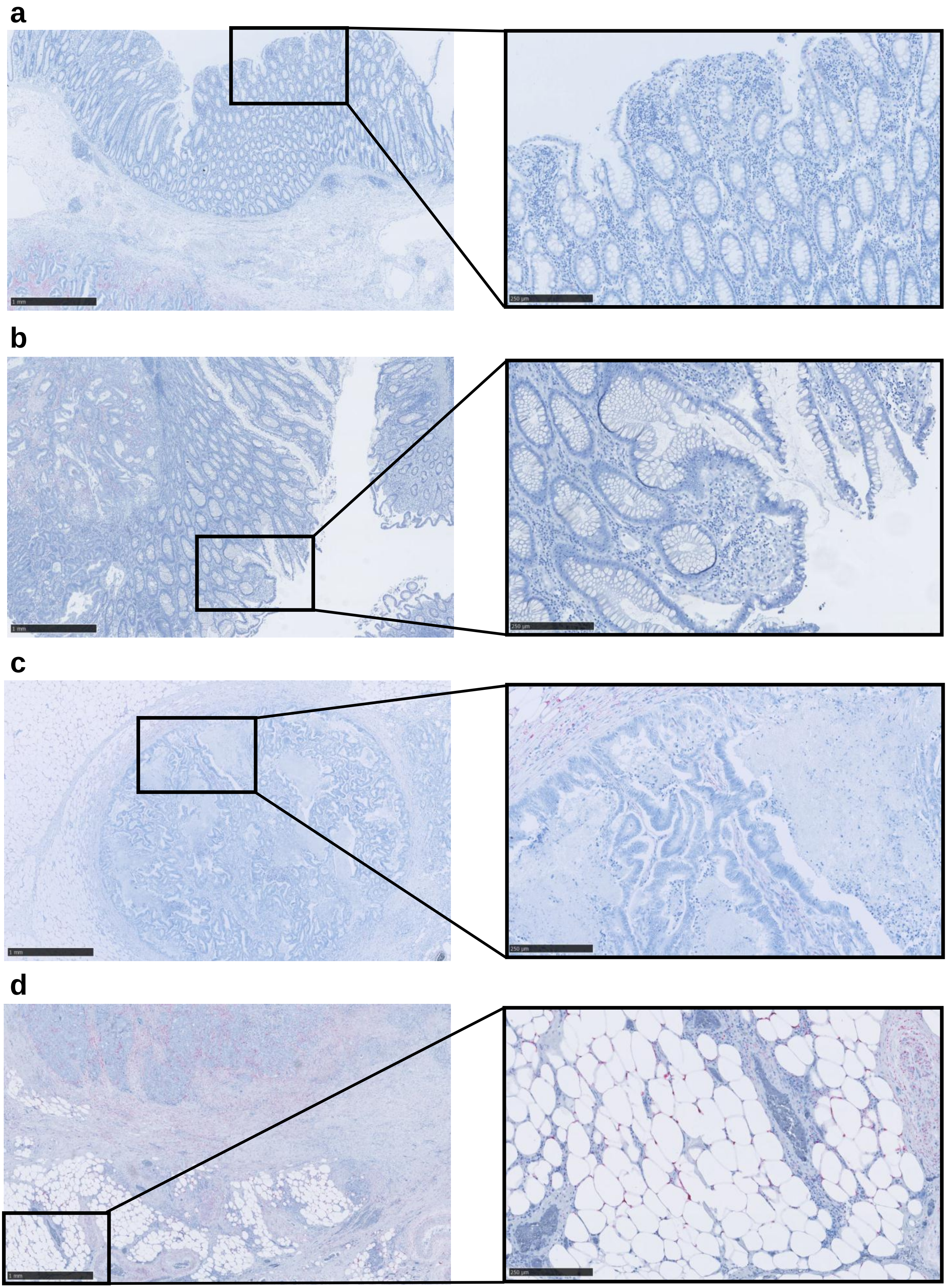
a
b
c
d

Supplementary Figure 1. RNA chromogenic *in situ* hybridization (CISH) for ciRS-7 in colon adenocarcinoma. a, ciRS-7 negativity in normal colon mucosa adjacent to a colon cancer. **b,** ciRS-7 negativity in colon adenoma. **c,** ciRS-7 negativity in cancer cells from a colon cancer lymph node metastasis whereas positivity is observed in the stromal cells. **d,** ciRS-7 positivity in adjacent adipose tissue. Overviews (left) and higher magnifications (right), indicated in the overview with a square, are shown. The ciRS-7 signal is observed as red dots. All 16 samples analyzed contained normal colon mucosa and adipose tissue and similar results were observed for all. In total, 5 samples from lymph node metastasis containing both cancer cells and tumor stroma were analyzed and showed similar results. More than 5 samples contained colon adenomas and all showed similar results. Scale bars, corresponding to 1000 µm (overviews) and 250 µm (higher magnifications), are indicated in the lower-left corners.

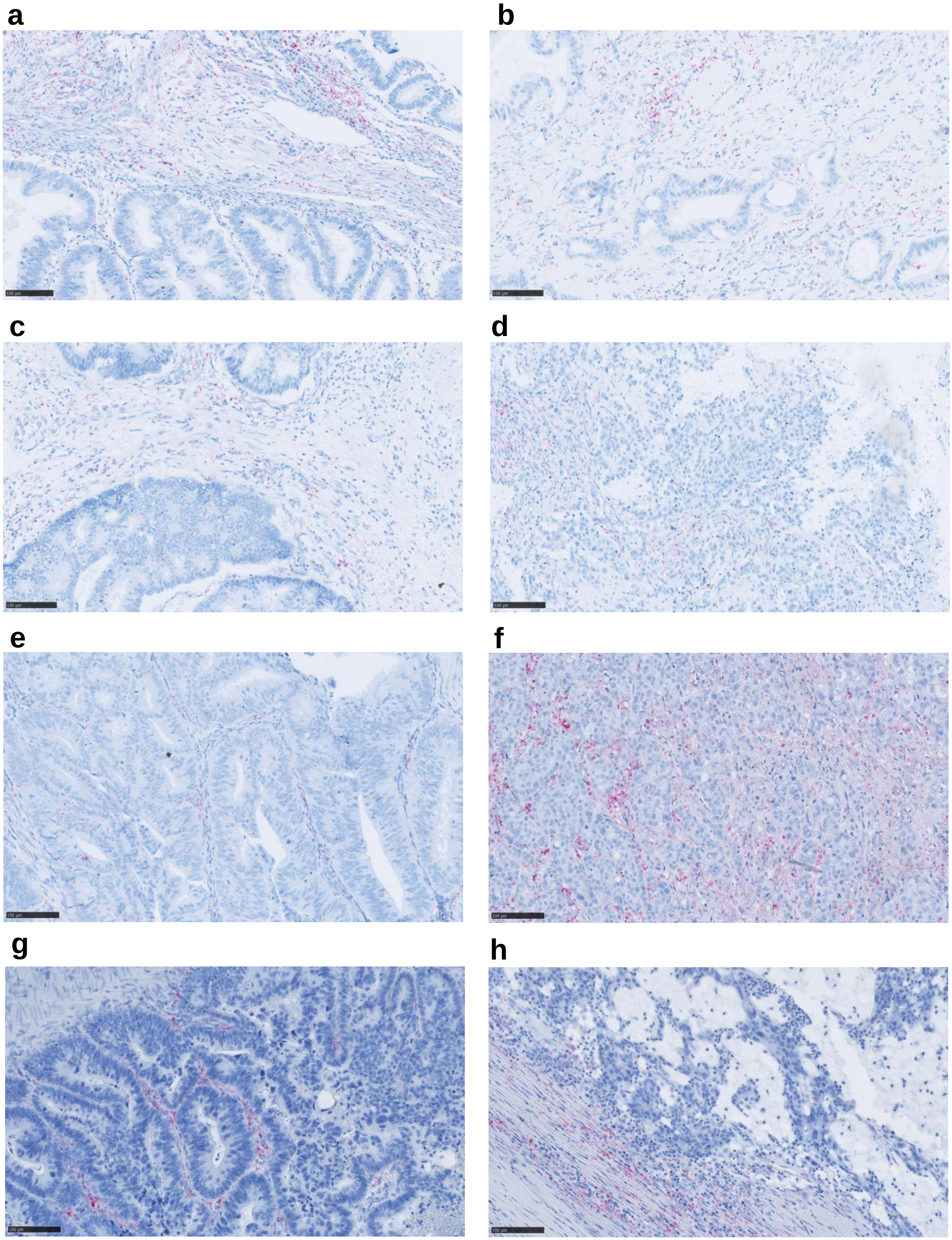
a
b
c
d
e
f
g
h

Supplementary Figure 2. RNA chromogenic *in situ* hybridization (CISH) for ciRS-7 in colon adenocarcinoma using TMAs. a-h, eight different representative colon cancer cores on a tissue microarray are shown. In total, 42 samples containing both cancer cells and tumor stroma were analyzed and showed similar results. The ciRS-7 signal (red dots) is observed in the tumor stroma whereas the cancer cells are negative. Scale bars, corresponding to 100 µm, are indicated in the lower-left corners.

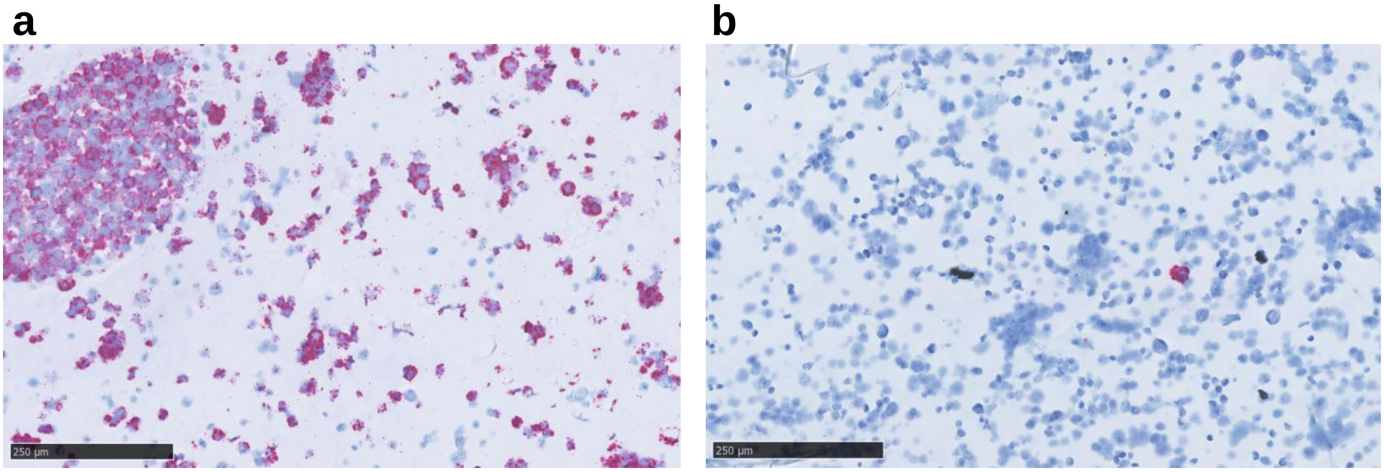

Supplementary Figure 3. RNA chromogenic *in situ* hybridization (CISH) for ciRS-7 in HEK293T ciRS-7 knock-out and wild-type cells. a, the ciRS-7 signal (red dots) is observed in the vast majority of the ciRS-7 wild-type cells. **b,** the ciRS-7 signal (red dots) is not observed in the ciRS-7 knock-out cells except in extremely rare cases. Since ciRS-7 is located on the X-chromosome and HEK293T cells contain three copies of the X chromosome, two of which are inactive, the rare expression of ciRS-7 are likely due to escape from X-chromosome inactivation. Scale bars corresponding to 250 µm, are indicated in the lower-left corners. This experiment was not repeated independently.

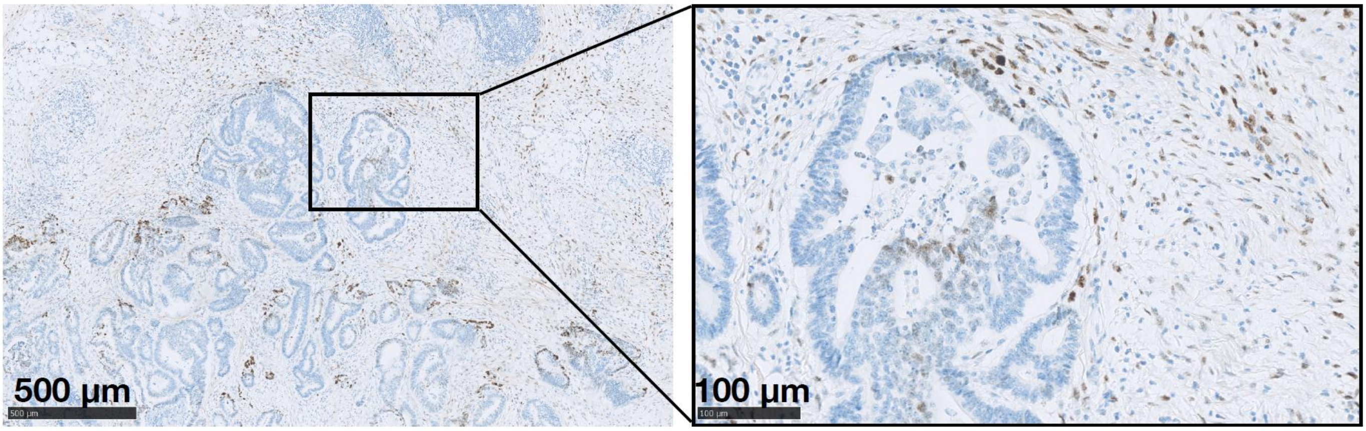

Supplementary Figure 4. FOS immunohistochemistry in colon adenocarcinoma. Immunohistochemistry analyses for FOS in a representative colon adenocarcinoma. Overview (left) and higher magnification (right), indicated in the overview with a square, are shown. The FOS signal (brown spots) is observed in the tumor stroma whereas the cancer cells are primarily negative. In total, 10 samples containing both cancer cells and tumor stroma were analyzed and showed similar results. Scale bars, corresponding to 500 µm (overviews) and 100 µm (higher magnifications), are indicated in the lower-left corners

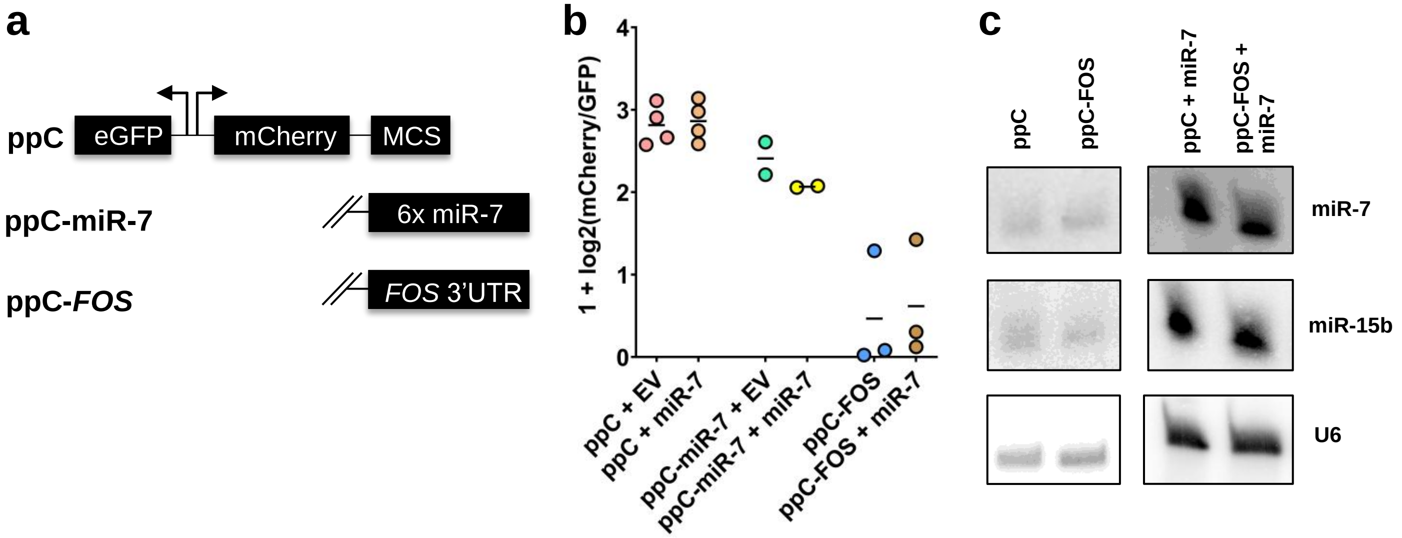

Supplementary Figure 5. Dual color *FOS* reporter assay in HEK293T cells. a, Schematic of dual color (ppC) plasmid constructs. // indicates that the sequence of the plasmid on the left hand of the schematic is identical to the construct listed at the top. **b,** Log2-normalized mCherry/GFP ratio quantified in ImageJ for each reporter with or without simultaneous co-overexpression of miR-7 in HEK293T cells (1:2 ratio). **c,** PAGE northern blot analysis of steady state levels of endogenous (left) or ectopically (right) expressed miR-7 upon (co-) transfection with the indicated dual-color reporter.

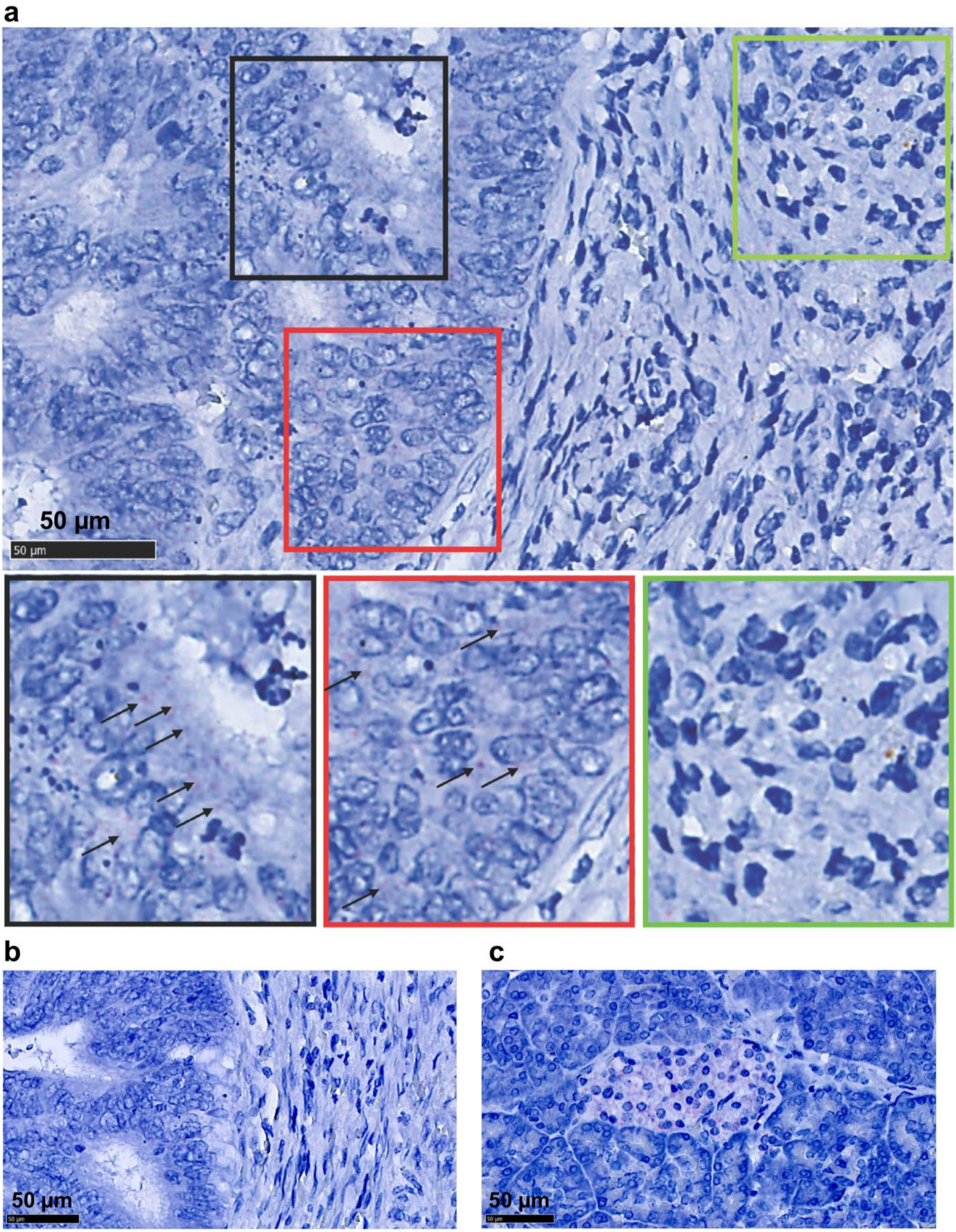
a
50 µm
50 µm
b
50 µm
50 µm
c
50 µm
50 µm

Supplementary Figure 6. miR-7 chromogenic *in situ* hybridization (CISH) in colon adenocarcinoma and control tissues. a, the miR-7 signal (red dots) is observed in colon cancer cells. Arrows are pointing to selected dots in the higher magnifications, which are indicated in the overview with color-coded squares (red and black). No miR-7 signal is observed in the tumor stroma. Higher magnification is indicated in the overview with green square. **b,** negative control using a scramble probe in colon adenocarcinoma. **c,** pancreatic tissue was used as a positive control as miR-7 is abundant in pancreatic islets. It can be observed that the signal (red dots) localize to the islets. In total, 4 colon cancer samples containing both cancer cells and tumor stroma were analyzed and showed similar results. The positive and negative controls were not repeated. Scale bars (50 µM) are indicated in the lower-left corners.

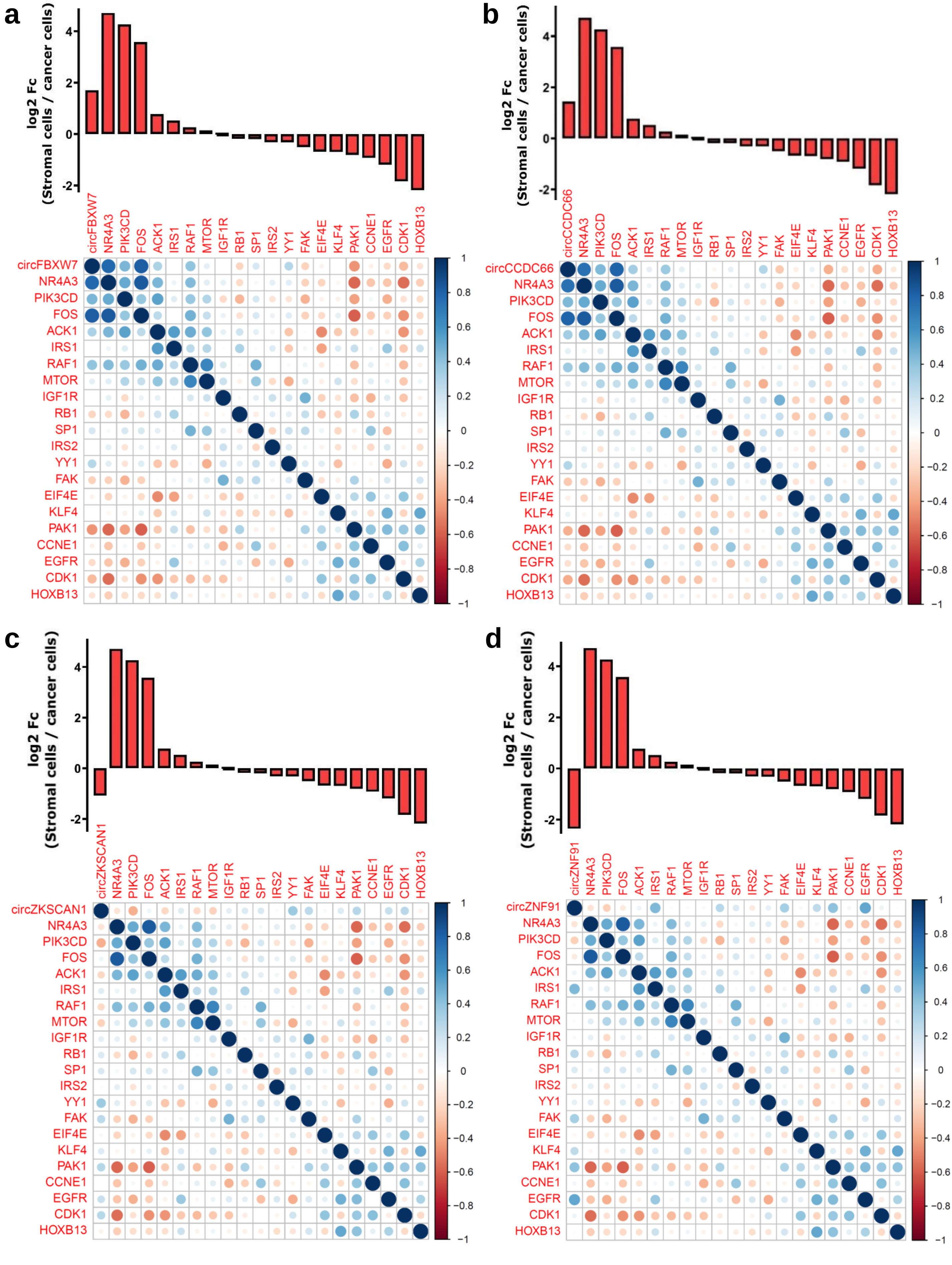
a
b
c
d
log2 Fc (Stromal cells / cancer cells)
circFBXW7
circCCDC66
circZKSCAN1
circZNF91
NR4A3
PIK3CD
FOS
ACK1
IRS1
RAF1
MTOR
IGF1R
RB1
SP1
IRS2
YY1
FAK
EIF4E
KLF4
PAK1
CCNE1
EGFR
CDK1
HOXB13

Supplementary Figure 7. Correlations between miR-7 target genes and circRNAs that do not contain miR-7 binding sites. a-d, Correlation matrixes showing Pearson correlation coefficients between circFBXW7 (**a**), circCCDC66 (**b**), circZKSCAN1 (**c**), and circZNF91 (**d**) and the 20 miR-7 target genes. The genes are listed according to relative expression levels in cancer and stromal cells, displayed in the top of each panel. These data were derived from fractions of stromal and cancer cells isolated by laser capture microdissection of colon cancer tissues, pooled from four individual representative patient samples (n=1).

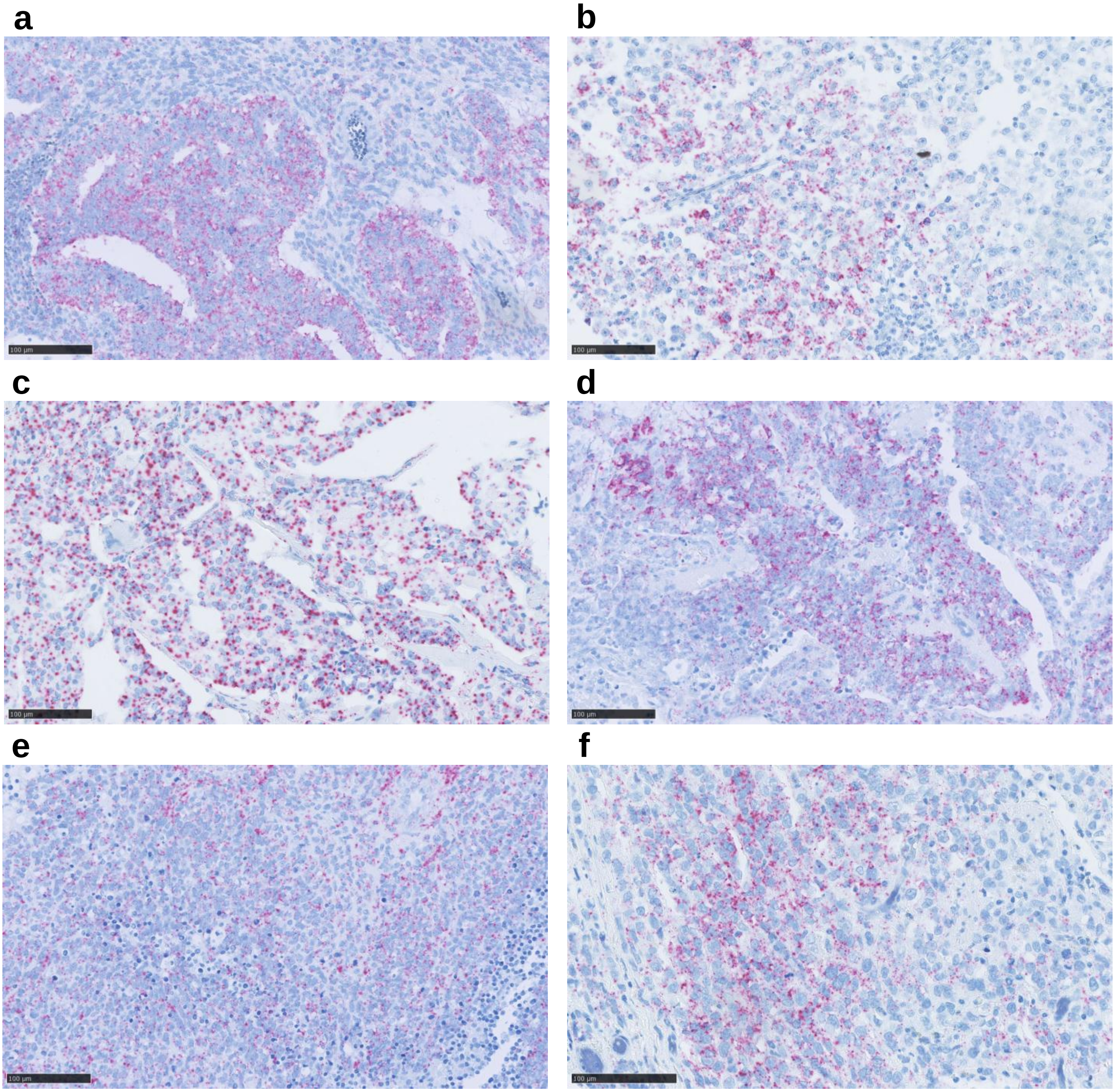

Supplementary Figure 8. RNA chromogenic in situ hybridization (CISH) for ciRS-7 in various malignant tumors. a-f, The pictures are derived from a tissue microarray and represent embryonal carcinoma **(a)**, seminoma **(b)**, medullary thyroid carcinoma **(c)**, neuroendocrine pancreatic carcinoma **(d)**, rhabdomyosarcoma **(e)** and malignant melanoma **(f)**. In contrast to the expression pattern observed in colon cancer and other adenocarcinomas, the ciRS-7 signal (red dots) is observed in the cancer cells as well as in the tumor stroma. For each cancer type at least two cores containing both cancer cells and tumor stroma were analyzed and showed similar results. Scale bars, corresponding to 100 µm, are indicated in the lower-left corners.
