# Supplementary material for: Spatial expression analyses of the putative oncogene ciRS-7 in cancer reshape the microRNA sponge theory
Source: Nat Commun. 2020 Sep 11;11:4551. doi: 10.1038/s41467-020-18355-2 (PMC7486402; doi:10.1038/s41467-020-18355-2)
Supplement: Supplementary file 3 — Description of Additional Supplementary Files [file 41467_2020_18355_MOESM3_ESM.pdf]

## Description of Additional Supplementary Files

File Name: Supplementary Data 1

Description: **miRNA expression in cancer and stromal cells.** Normalized NanoString nCounter expression data for 799 miRNAs in fractions of cancer and stromal cells isolated by laser capture microdissection of colon cancer LCMD tissues, pooled from four individual representative patient samples (n=1).
