# Supplementary material for: Spatial expression analyses of the putative oncogene ciRS-7 in cancer reshape the microRNA sponge theory
Source: Nat Commun. 2020 Sep 11;11:4551. doi: 10.1038/s41467-020-18355-2 (PMC7486402; doi:10.1038/s41467-020-18355-2)
Supplement: Supplementary file 4 — Supplementary Data 1 [file 41467_2020_18355_MOESM4_ESM.docx]

**Supplementary Data 1. Normalized NanoString nCounter miRNA data in cancer- and stromal cell fractions.**

| **Targeted miRNA(s)** | **Cancer cells** | **Stromal cells** | **Total expression** | **Fold change** |
| --- | --- | --- | --- | --- |
| hsa-miR-4454 +  hsa-miR-7975 | 101459.5 | 114177.02 | 215636.5 | 0.888616 |
| hsa-miR-4286 | 10686.08 | 3406.88 | 14092.96 | 3.136618 |
| hsa-miR-494-3p | 4360.6 | 2581.1 | 6941.7 | 1.689435 |
| hsa-let-7a-5p | 3764.7 | 1055.16 | 4819.86 | 3.567895 |
| hsa-miR-29b-3p | 2839.61 | 109.23 | 2948.84 | 25.99661 |
| hsa-miR-21-5p | 2693.2 | 543.97 | 3237.17 | 4.951008 |
| hsa-let-7b-5p | 2200.82 | 1437.47 | 3638.29 | 1.531037 |
| hsa-miR-194-5p | 1686.98 | 37.14 | 1724.12 | 45.42219 |
| hsa-miR-200c-3p | 1682.32 | 62.26 | 1744.58 | 27.02088 |
| hsa-miR-200b-3p | 1518.19 | 65.54 | 1583.73 | 23.16433 |
| hsa-miR-1285-5p | 1142.37 | 1263.79 | 2406.16 | 0.903924 |
| hsa-miR-23a-3p | 1071.5 | 435.83 | 1507.33 | 2.458527 |
| hsa-let-7g-5p | 1063.11 | 166.03 | 1229.14 | 6.40312 |
| hsa-miR-148a-3p | 943.74 | 116.88 | 1060.62 | 8.074435 |
| hsa-miR-29a-3p | 940.01 | 108.14 | 1048.15 | 8.692528 |
| hsa-miR-141-3p | 864.47 | 92.85 | 957.32 | 9.310393 |
| hsa-miR-16-5p | 780.54 | 148.55 | 929.09 | 5.254392 |
| hsa-let-7i-5p | 761.89 | 358.27 | 1120.16 | 2.126581 |
| hsa-miR-1972 | 734.85 | 995.09 | 1729.94 | 0.738476 |
| hsa-miR-20a-5p +  hsa-miR-20b-5p | 705.01 | 33.86 | 738.87 | 20.82132 |
| hsa-miR-93-5p | 667.71 | 50.25 | 717.96 | 13.28776 |
| hsa-miR-200a-3p | 626.67 | 64.45 | 691.12 | 9.723351 |
| hsa-miR-106a-5p +  hsa-miR-17-5p | 610.82 | 49.15 | 659.97 | 12.42767 |
| hsa-miR-302d-3p | 578.18 | 704.53 | 1282.71 | 0.820661 |
| hsa-miR-4516 | 555.8 | 389.95 | 945.75 | 1.425311 |
| hsa-miR-222-3p | 541.81 | 115.78 | 657.59 | 4.679651 |
| hsa-miR-10a-5p | 540.88 | 75.37 | 616.25 | 7.17633 |
| hsa-miR-181a-5p | 514.77 | 217.37 | 732.14 | 2.368174 |
| hsa-miR-1246 | 513.83 | 139.81 | 653.64 | 3.675202 |
| hsa-miR-135b-5p | 503.58 | 43.69 | 547.27 | 11.52621 |
| hsa-miR-1253 | 476.53 | 452.21 | 928.74 | 1.05378 |
| hsa-miR-191-5p | 454.15 | 143.09 | 597.24 | 3.173877 |
| hsa-miR-630 | 437.37 | 323.32 | 760.69 | 1.352747 |
| hsa-miR-126-3p | 429.91 | 156.2 | 586.11 | 2.752305 |
| hsa-miR-574-5p | 427.11 | 257.78 | 684.89 | 1.656878 |
| hsa-miR-25-3p | 425.24 | 55.71 | 480.95 | 7.6331 |
| hsa-miR-374a-5p | 388.87 | 99.4 | 488.27 | 3.912173 |
| hsa-miR-196b-5p | 387.94 | 58.98 | 446.92 | 6.577484 |
| hsa-let-7d-5p | 385.14 | 96.12 | 481.26 | 4.006866 |
| hsa-miR-612 | 366.49 | 431.46 | 797.95 | 0.849418 |
| hsa-miR-579-3p | 361.83 | 1.09 | 362.92 | 331.9541 |
| hsa-miR-4488 | 350.64 | 292.74 | 643.38 | 1.197786 |
| hsa-miR-15b-5p | 312.4 | 92.85 | 405.25 | 3.364567 |
| hsa-miR-1283 | 285.36 | 361.55 | 646.91 | 0.789268 |
| hsa-miR-19b-3p | 272.3 | 22.94 | 295.24 | 11.8701 |
| hsa-miR-142-3p | 265.78 | 103.77 | 369.55 | 2.561241 |
| hsa-miR-1260a | 263.91 | 292.74 | 556.65 | 0.901517 |
| hsa-miR-15a-5p | 238.73 | 40.42 | 279.15 | 5.906235 |
| hsa-miR-106b-5p | 234.07 | 41.51 | 275.58 | 5.638882 |
| hsa-miR-199a-3p +  hsa-miR-199b-3p | 231.27 | 509.01 | 740.28 | 0.454353 |
| hsa-miR-575 | 218.22 | 137.63 | 355.85 | 1.585555 |
| hsa-miR-19a-3p | 215.42 | 50.25 | 265.67 | 4.286965 |
| hsa-miR-146a-5p | 207.96 | 63.35 | 271.31 | 3.282715 |
| hsa-miR-203a-3p | 202.36 | 56.8 | 259.16 | 3.562676 |
| hsa-miR-320e | 200.5 | 182.41 | 382.91 | 1.099172 |
| hsa-miR-26a-5p | 200.5 | 97.21 | 297.71 | 2.062545 |
| hsa-miR-18a-5p | 198.63 | 58.98 | 257.61 | 3.367752 |
| hsa-miR-7-5p | 195.84 | 40.42 | 236.26 | 4.845126 |
| hsa-miR-378e | 192.11 | 207.54 | 399.65 | 0.925653 |
| hsa-miR-196a-5p | 192.11 | 43.69 | 235.8 | 4.397116 |
| hsa-miR-363-3p | 189.31 | 243.58 | 432.89 | 0.777198 |
| hsa-miR-192-5p | 189.31 | 38.23 | 227.54 | 4.95187 |
| hsa-miR-125b-5p | 187.44 | 924.09 | 1111.53 | 0.202837 |
| hsa-miR-6721-5p | 185.58 | 243.58 | 429.16 | 0.761885 |
| hsa-miR-548ar-5p | 184.64 | 239.21 | 423.85 | 0.771874 |
| hsa-miR-361-5p | 182.78 | 95.03 | 277.81 | 1.923393 |
| hsa-miR-22-3p | 180.91 | 111.41 | 292.32 | 1.623822 |
| hsa-miR-34a-5p | 176.25 | 86.29 | 262.54 | 2.042531 |
| hsa-let-7f-5p | 175.32 | 56.8 | 232.12 | 3.08662 |
| hsa-miR-145-5p | 171.59 | 762.43 | 934.02 | 0.225057 |
| hsa-let-7c-5p | 168.79 | 192.24 | 361.03 | 0.878017 |
| hsa-miR-199a-5p | 167.86 | 367.01 | 534.87 | 0.457372 |
| hsa-miR-873-3p | 165.06 | 222.83 | 387.89 | 0.740744 |
| hsa-miR-598-3p | 161.33 | 191.15 | 352.48 | 0.843997 |
| hsa-miR-888-5p | 161.33 | 138.72 | 300.05 | 1.16299 |
| hsa-miR-107 | 157.6 | 79.74 | 237.34 | 1.976423 |
| hsa-miR-26b-5p | 156.67 | 52.43 | 209.1 | 2.988175 |
| hsa-miR-451a | 152.94 | 108.14 | 261.08 | 1.414278 |
| hsa-miR-27b-3p | 148.28 | 72.09 | 220.37 | 2.056873 |
| hsa-miR-215-5p | 146.41 | 81.92 | 228.33 | 1.787231 |
| hsa-miR-98-5p | 146.41 | 54.61 | 201.02 | 2.681011 |
| hsa-miR-24-3p | 146.41 | 49.15 | 195.56 | 2.97884 |
| hsa-miR-495-3p | 143.61 | 167.12 | 310.73 | 0.859323 |
| hsa-miR-23b-3p | 142.68 | 108.14 | 250.82 | 1.319401 |
| hsa-miR-95-3p | 137.08 | 81.92 | 219 | 1.67334 |
| hsa-let-7e-5p | 135.22 | 77.55 | 212.77 | 1.743649 |
| hsa-miR-1469 | 125.89 | 66.63 | 192.52 | 1.889389 |
| hsa-miR-28-5p | 124.96 | 99.4 | 224.36 | 1.257143 |
| hsa-miR-155-5p | 124.03 | 132.17 | 256.2 | 0.938413 |
| hsa-miR-221-3p | 121.23 | 49.15 | 170.38 | 2.466531 |
| hsa-miR-3144-3p | 119.37 | 138.72 | 258.09 | 0.86051 |
| hsa-miR-423-5p | 118.43 | 115.78 | 234.21 | 1.022888 |
| hsa-miR-378i | 115.64 | 78.65 | 194.29 | 1.470312 |
| hsa-miR-30e-5p | 114.7 | 68.81 | 183.51 | 1.666909 |
| hsa-miR-429 | 113.77 | 34.95 | 148.72 | 3.255222 |
| hsa-miR-186-5p | 112.84 | 95.03 | 207.87 | 1.187415 |
| hsa-miR-223-3p | 112.84 | 84.11 | 196.95 | 1.341577 |
| hsa-miR-1915-3p | 111.91 | 115.78 | 227.69 | 0.966575 |
| hsa-miR-877-5p | 111.91 | 92.85 | 204.76 | 1.205277 |
| hsa-miR-4455 | 110.97 | 112.51 | 223.48 | 0.986312 |
| hsa-miR-1276 | 110.97 | 99.4 | 210.37 | 1.116398 |
| hsa-miR-664a-3p | 110.04 | 176.95 | 286.99 | 0.621871 |
| hsa-miR-4443 | 110.04 | 120.15 | 230.19 | 0.915855 |
| hsa-miR-548ah-5p | 109.11 | 114.69 | 223.8 | 0.951347 |
| hsa-miR-206 | 109.11 | 84.11 | 193.22 | 1.29723 |
| hsa-miR-1973 | 109.11 | 68.81 | 177.92 | 1.585671 |
| hsa-miR-374b-5p | 109.11 | 57.89 | 167 | 1.884781 |
| hsa-miR-593-3p | 104.45 | 105.95 | 210.4 | 0.985842 |
| hsa-miR-375 | 102.58 | 50.25 | 152.83 | 2.041393 |
| hsa-miR-130a-3p | 101.65 | 190.06 | 291.71 | 0.534831 |
| hsa-miR-592 | 100.72 | 68.81 | 169.53 | 1.463741 |
| hsa-miR-125a-5p | 99.78 | 120.15 | 219.93 | 0.830462 |
| hsa-miR-30d-5p | 96.05 | 50.25 | 146.3 | 1.911443 |
| hsa-miR-199b-5p | 94.19 | 121.25 | 215.44 | 0.776825 |
| hsa-miR-548d-3p | 94.19 | 71 | 165.19 | 1.32662 |
| hsa-miR-548n | 92.32 | 65.54 | 157.86 | 1.408605 |
| hsa-miR-183-5p | 91.39 | 86.29 | 177.68 | 1.059103 |
| hsa-miR-371a-5p | 91.39 | 71 | 162.39 | 1.287183 |
| hsa-miR-1290 | 90.46 | 83.01 | 173.47 | 1.089748 |
| hsa-miR-150-5p | 89.52 | 300.38 | 389.9 | 0.298023 |
| hsa-miR-603 | 86.73 | 90.66 | 177.39 | 0.956651 |
| hsa-miR-548aa +  hsa-miR-548t-3p | 86.73 | 85.2 | 171.93 | 1.017958 |
| hsa-miR-1183 | 84.86 | 137.63 | 222.49 | 0.616581 |
| hsa-miR-664b-3p | 84.86 | 108.14 | 193 | 0.784724 |
| hsa-miR-365a-3p +  hsa-miR-365b-3p | 84.86 | 96.12 | 180.98 | 0.882855 |
| hsa-miR-181b-5p +  hsa-miR-181d-5p | 84.86 | 45.88 | 130.74 | 1.849608 |
| hsa-miR-1200 | 83.93 | 89.57 | 173.5 | 0.937032 |
| hsa-miR-425-5p | 83.93 | 34.95 | 118.88 | 2.401431 |
| hsa-miR-1323 | 82.06 | 110.32 | 192.38 | 0.743836 |
| hsa-miR-2117 | 82.06 | 83.01 | 165.07 | 0.988556 |
| hsa-miR-644a | 82.06 | 79.74 | 161.8 | 1.029095 |
| hsa-miR-582-5p | 82.06 | 77.55 | 159.61 | 1.058156 |
| hsa-miR-511-5p | 82.06 | 75.37 | 157.43 | 1.088762 |
| hsa-miR-629-5p | 82.06 | 68.81 | 150.87 | 1.192559 |
| hsa-miR-663a | 82.06 | 64.45 | 146.51 | 1.273235 |
| hsa-miR-143-3p | 81.13 | 122.34 | 203.47 | 0.663152 |
| hsa-miR-132-3p | 81.13 | 65.54 | 146.67 | 1.23787 |
| hsa-miR-10b-5p | 80.2 | 58.98 | 139.18 | 1.359783 |
| hsa-miR-29c-3p | 80.2 | 40.42 | 120.62 | 1.984166 |
| hsa-miR-642a-3p | 79.27 | 88.48 | 167.75 | 0.895909 |
| hsa-miR-1279 | 79.27 | 79.74 | 159.01 | 0.994106 |
| hsa-miR-28-3p | 79.27 | 66.63 | 145.9 | 1.189704 |
| hsa-miR-100-5p | 78.33 | 173.68 | 252.01 | 0.451002 |
| hsa-miR-1297 | 78.33 | 85.2 | 163.53 | 0.919366 |
| hsa-miR-4284 | 78.33 | 62.26 | 140.59 | 1.258111 |
| hsa-miR-922 | 77.4 | 74.28 | 151.68 | 1.042003 |
| hsa-miR-1303 | 76.47 | 88.48 | 164.95 | 0.864263 |
| hsa-miR-362-5p | 76.47 | 72.09 | 148.56 | 1.060757 |
| hsa-miR-4532 | 76.47 | 48.06 | 124.53 | 1.591136 |
| hsa-miR-517a-3p | 75.54 | 91.75 | 167.29 | 0.823324 |
| hsa-miR-30b-5p | 75.54 | 48.06 | 123.6 | 1.571785 |
| hsa-miR-99b-5p | 74.6 | 99.4 | 174 | 0.750503 |
| hsa-miR-891b | 73.67 | 97.21 | 170.88 | 0.757844 |
| hsa-miR-324-3p | 72.74 | 74.28 | 147.02 | 0.979268 |
| hsa-miR-324-5p | 72.74 | 66.63 | 139.37 | 1.0917 |
| hsa-miR-502-5p | 72.74 | 58.98 | 131.72 | 1.233299 |
| hsa-miR-137 | 71.81 | 101.58 | 173.39 | 0.70693 |
| hsa-miR-585-3p | 71.81 | 78.65 | 150.46 | 0.913032 |
| hsa-miR-146b-5p | 70.87 | 76.46 | 147.33 | 0.92689 |
| hsa-miR-181c-5p | 70.87 | 63.35 | 134.22 | 1.118706 |
| hsa-miR-590-5p | 69.94 | 76.46 | 146.4 | 0.914727 |
| hsa-miR-378g | 69.94 | 71 | 140.94 | 0.98507 |
| hsa-miR-454-3p | 69.94 | 65.54 | 135.48 | 1.067135 |
| hsa-miR-3180-3p | 69.01 | 83.01 | 152.02 | 0.831346 |
| hsa-miR-4755-5p | 69.01 | 62.26 | 131.27 | 1.108416 |
| hsa-miR-627-5p | 67.14 | 96.12 | 163.26 | 0.698502 |
| hsa-miR-299-5p | 67.14 | 92.85 | 159.99 | 0.723102 |
| hsa-miR-379-5p | 67.14 | 75.37 | 142.51 | 0.890805 |
| hsa-miR-299-3p | 67.14 | 71 | 138.14 | 0.945634 |
| hsa-miR-1305 | 67.14 | 67.72 | 134.86 | 0.991435 |
| hsa-miR-656-3p | 67.14 | 60.08 | 127.22 | 1.11751 |
| hsa-miR-205-5p | 67.14 | 39.32 | 106.46 | 1.707528 |
| hsa-miR-342-3p | 66.21 | 143.09 | 209.3 | 0.462716 |
| hsa-miR-493-3p | 66.21 | 65.54 | 131.75 | 1.010223 |
| hsa-miR-133a-3p | 64.35 | 104.86 | 169.21 | 0.613675 |
| hsa-miR-939-5p | 64.35 | 85.2 | 149.55 | 0.755282 |
| hsa-miR-548v | 64.35 | 74.28 | 138.63 | 0.866317 |
| hsa-miR-216b-5p | 64.35 | 72.09 | 136.44 | 0.892634 |
| hsa-miR-421 | 64.35 | 69.91 | 134.26 | 0.920469 |
| hsa-miR-651-5p | 64.35 | 68.81 | 133.16 | 0.935184 |
| hsa-miR-301a-3p | 64.35 | 63.35 | 127.7 | 1.015785 |
| hsa-miR-548d-5p | 64.35 | 48.06 | 112.41 | 1.338951 |
| hsa-miR-539-5p | 64.35 | 42.6 | 106.95 | 1.510563 |
| hsa-miR-301b-3p | 63.41 | 80.83 | 144.24 | 0.784486 |
| hsa-miR-548z +  hsa-miR-548h-3p | 63.41 | 79.74 | 143.15 | 0.795209 |
| hsa-miR-489-3p | 63.41 | 69.91 | 133.32 | 0.907023 |
| hsa-miR-197-5p | 62.48 | 85.2 | 147.68 | 0.733333 |
| hsa-miR-548m | 62.48 | 80.83 | 143.31 | 0.77298 |
| hsa-miR-378h | 62.48 | 74.28 | 136.76 | 0.841142 |
| hsa-miR-30a-5p | 62.48 | 60.08 | 122.56 | 1.039947 |
| hsa-miR-1180-3p | 62.48 | 56.8 | 119.28 | 1.1 |
| hsa-miR-185-5p | 62.48 | 50.25 | 112.73 | 1.243383 |
| hsa-miR-4448 | 62.48 | 50.25 | 112.73 | 1.243383 |
| hsa-miR-149-5p | 61.55 | 83.01 | 144.56 | 0.741477 |
| hsa-miR-487a-3p | 61.55 | 81.92 | 143.47 | 0.751343 |
| hsa-miR-216a-5p | 61.55 | 79.74 | 141.29 | 0.771884 |
| hsa-miR-376a-3p | 61.55 | 73.18 | 134.73 | 0.841077 |
| hsa-miR-1206 | 61.55 | 65.54 | 127.09 | 0.939121 |
| hsa-miR-151a-3p | 61.55 | 63.35 | 124.9 | 0.971586 |
| hsa-miR-377-3p | 61.55 | 58.98 | 120.53 | 1.043574 |
| hsa-miR-1307-3p | 61.55 | 53.52 | 115.07 | 1.150037 |
| hsa-miR-1295a | 61.55 | 51.34 | 112.89 | 1.19887 |
| hsa-miR-4485-3p | 61.55 | 51.34 | 112.89 | 1.19887 |
| hsa-miR-876-3p | 61.55 | 51.34 | 112.89 | 1.19887 |
| hsa-miR-4536-5p | 60.62 | 81.92 | 142.54 | 0.73999 |
| hsa-miR-761 | 60.62 | 75.37 | 135.99 | 0.804299 |
| hsa-miR-1281 | 60.62 | 69.91 | 130.53 | 0.867115 |
| hsa-miR-1277-3p | 60.62 | 65.54 | 126.16 | 0.924931 |
| hsa-miR-4531 | 60.62 | 58.98 | 119.6 | 1.027806 |
| hsa-miR-608 | 60.62 | 55.71 | 116.33 | 1.088135 |
| hsa-miR-140-5p | 60.62 | 50.25 | 110.87 | 1.206368 |
| hsa-miR-639 | 59.68 | 93.94 | 153.62 | 0.635299 |
| hsa-miR-23c | 59.68 | 86.29 | 145.97 | 0.691621 |
| hsa-miR-497-5p | 59.68 | 81.92 | 141.6 | 0.728516 |
| hsa-miR-411-5p | 59.68 | 77.55 | 137.23 | 0.769568 |
| hsa-miR-331-3p | 59.68 | 68.81 | 128.49 | 0.867316 |
| hsa-miR-3161 | 59.68 | 63.35 | 123.03 | 0.942068 |
| hsa-miR-212-3p | 59.68 | 62.26 | 121.94 | 0.958561 |
| hsa-miR-127-5p | 59.68 | 53.52 | 113.2 | 1.115097 |
| hsa-miR-891a-5p | 58.75 | 98.31 | 157.06 | 0.597599 |
| hsa-miR-346 | 58.75 | 66.63 | 125.38 | 0.881735 |
| hsa-miR-1197 | 58.75 | 65.54 | 124.29 | 0.896399 |
| hsa-miR-337-3p | 58.75 | 58.98 | 117.73 | 0.9961 |
| hsa-miR-197-3p | 58.75 | 56.8 | 115.55 | 1.034331 |
| hsa-miR-769-5p | 58.75 | 53.52 | 112.27 | 1.09772 |
| hsa-miR-361-3p | 57.82 | 62.26 | 120.08 | 0.928686 |
| hsa-miR-548i | 57.82 | 58.98 | 116.8 | 0.980332 |
| hsa-miR-526b-5p | 57.82 | 50.25 | 108.07 | 1.150647 |
| hsa-miR-640 | 57.82 | 42.6 | 100.42 | 1.357277 |
| hsa-miR-597-5p | 56.89 | 75.37 | 132.26 | 0.75481 |
| hsa-miR-3614-5p | 56.89 | 68.81 | 125.7 | 0.826769 |
| hsa-miR-577 | 56.89 | 64.45 | 121.34 | 0.8827 |
| hsa-miR-301b-5p | 56.89 | 63.35 | 120.24 | 0.898027 |
| hsa-miR-3185 | 56.89 | 61.17 | 118.06 | 0.930031 |
| hsa-miR-30e-3p | 56.89 | 60.08 | 116.97 | 0.946904 |
| hsa-miR-615-3p | 56.89 | 57.89 | 114.78 | 0.982726 |
| hsa-miR-335-5p | 56.89 | 54.61 | 111.5 | 1.041751 |
| hsa-miR-548q | 56.89 | 48.06 | 104.95 | 1.183729 |
| hsa-miR-4451 | 55.95 | 67.72 | 123.67 | 0.826196 |
| hsa-miR-378f | 55.95 | 63.35 | 119.3 | 0.883189 |
| hsa-miR-589-5p | 55.95 | 57.89 | 113.84 | 0.966488 |
| hsa-miR-196a-3p | 55.95 | 55.71 | 111.66 | 1.004308 |
| hsa-miR-502-3p | 55.95 | 55.71 | 111.66 | 1.004308 |
| hsa-miR-4431 | 55.02 | 74.28 | 129.3 | 0.740711 |
| hsa-miR-548ar-3p | 55.02 | 63.35 | 118.37 | 0.868508 |
| hsa-miR-1322 | 55.02 | 61.17 | 116.19 | 0.899461 |
| hsa-miR-933 | 55.02 | 58.98 | 114 | 0.932859 |
| hsa-miR-298 | 55.02 | 54.61 | 109.63 | 1.007508 |
| hsa-miR-625-5p | 55.02 | 40.42 | 95.44 | 1.361207 |
| hsa-miR-188-5p | 54.09 | 76.46 | 130.55 | 0.707429 |
| hsa-miR-548al | 54.09 | 74.28 | 128.37 | 0.728191 |
| hsa-miR-211-5p | 54.09 | 72.09 | 126.18 | 0.750312 |
| hsa-miR-147a | 54.09 | 69.91 | 124 | 0.773709 |
| hsa-miR-1296-3p | 54.09 | 64.45 | 118.54 | 0.839255 |
| hsa-miR-371b-5p | 54.09 | 62.26 | 116.35 | 0.868776 |
| hsa-miR-182-3p | 54.09 | 58.98 | 113.07 | 0.917091 |
| hsa-miR-496 | 54.09 | 53.52 | 107.61 | 1.01065 |
| hsa-miR-219a-1-3p | 54.09 | 50.25 | 104.34 | 1.076418 |
| hsa-miR-765 | 54.09 | 49.15 | 103.24 | 1.100509 |
| hsa-miR-148b-3p | 54.09 | 48.06 | 102.15 | 1.125468 |
| hsa-miR-572 | 54.09 | 48.06 | 102.15 | 1.125468 |
| hsa-miR-1257 | 54.09 | 44.78 | 98.87 | 1.207905 |
| hsa-miR-340-5p | 54.09 | 36.05 | 90.14 | 1.500416 |
| hsa-miR-3147 | 53.16 | 73.18 | 126.34 | 0.726428 |
| hsa-miR-1245b-5p | 53.16 | 65.54 | 118.7 | 0.811108 |
| hsa-miR-128-1-5p | 53.16 | 46.97 | 100.13 | 1.131786 |
| hsa-miR-182-5p | 53.16 | 44.78 | 97.94 | 1.187137 |
| hsa-miR-4741 | 53.16 | 43.69 | 96.85 | 1.216754 |
| hsa-miR-548k | 53.16 | 42.6 | 95.76 | 1.247887 |
| hsa-miR-135a-5p | 53.16 | 38.23 | 91.39 | 1.390531 |
| hsa-miR-548a-5p | 52.22 | 73.18 | 125.4 | 0.713583 |
| hsa-miR-503-5p | 52.22 | 72.09 | 124.31 | 0.724372 |
| hsa-miR-495-5p | 52.22 | 71 | 123.22 | 0.735493 |
| hsa-miR-764 | 52.22 | 71 | 123.22 | 0.735493 |
| hsa-miR-499a-5p | 52.22 | 64.45 | 116.67 | 0.81024 |
| hsa-miR-424-5p | 52.22 | 63.35 | 115.57 | 0.824309 |
| hsa-miR-2682-5p | 52.22 | 61.17 | 113.39 | 0.853686 |
| hsa-miR-1270 | 52.22 | 56.8 | 109.02 | 0.919366 |
| hsa-miR-514b-5p | 52.22 | 53.52 | 105.74 | 0.97571 |
| hsa-miR-525-5p | 52.22 | 52.43 | 104.65 | 0.995995 |
| hsa-miR-1286 | 52.22 | 45.88 | 98.1 | 1.138187 |
| hsa-miR-25-5p | 51.29 | 74.28 | 125.57 | 0.690495 |
| hsa-miR-1306-3p | 51.29 | 63.35 | 114.64 | 0.809629 |
| hsa-miR-648 | 51.29 | 58.98 | 110.27 | 0.869617 |
| hsa-miR-542-3p | 51.29 | 57.89 | 109.18 | 0.885991 |
| hsa-miR-600 | 51.29 | 41.51 | 92.8 | 1.235606 |
| hsa-miR-218-5p | 51.29 | 38.23 | 89.52 | 1.341617 |
| hsa-miR-660-5p | 51.29 | 29.49 | 80.78 | 1.739234 |
| hsa-miR-744-5p | 50.36 | 68.81 | 119.17 | 0.73187 |
| hsa-miR-1249-3p | 50.36 | 67.72 | 118.08 | 0.74365 |
| hsa-miR-1228-3p | 50.36 | 56.8 | 107.16 | 0.88662 |
| hsa-miR-152-3p | 50.36 | 55.71 | 106.07 | 0.903967 |
| hsa-miR-128-3p | 50.36 | 54.61 | 104.97 | 0.922175 |
| hsa-miR-509-3-5p | 50.36 | 51.34 | 101.7 | 0.980912 |
| hsa-miR-136-5p | 50.36 | 49.15 | 99.51 | 1.024619 |
| hsa-miR-134-5p +  hsa-miR-6728-5p | 50.36 | 48.06 | 98.42 | 1.047857 |
| hsa-miR-345-3p | 50.36 | 48.06 | 98.42 | 1.047857 |
| hsa-miR-302f | 50.36 | 39.32 | 89.68 | 1.280773 |
| hsa-miR-151a-5p | 50.36 | 29.49 | 79.85 | 1.707698 |
| hsa-miR-188-3p | 49.43 | 69.91 | 119.34 | 0.707052 |
| hsa-miR-543 | 49.43 | 66.63 | 116.06 | 0.741858 |
| hsa-miR-455-3p | 49.43 | 61.17 | 110.6 | 0.808076 |
| hsa-miR-1255b-5p | 49.43 | 60.08 | 109.51 | 0.822736 |
| hsa-miR-1224-3p | 49.43 | 54.61 | 104.04 | 0.905146 |
| hsa-miR-1304-5p | 49.43 | 50.25 | 99.68 | 0.983682 |
| hsa-miR-624-3p | 49.43 | 49.15 | 98.58 | 1.005697 |
| hsa-miR-890 | 49.43 | 49.15 | 98.58 | 1.005697 |
| hsa-miR-2113 | 49.43 | 46.97 | 96.4 | 1.052374 |
| hsa-miR-27a-3p | 49.43 | 39.32 | 88.75 | 1.257121 |
| hsa-miR-92a-1-5p | 49.43 | 33.86 | 83.29 | 1.459835 |
| hsa-miR-181d-3p | 48.49 | 87.38 | 135.87 | 0.554932 |
| hsa-miR-302e | 48.49 | 68.81 | 117.3 | 0.704694 |
| hsa-miR-337-5p | 48.49 | 68.81 | 117.3 | 0.704694 |
| hsa-miR-1537-3p | 48.49 | 67.72 | 116.21 | 0.716037 |
| hsa-miR-5010-3p | 48.49 | 61.17 | 109.66 | 0.792709 |
| hsa-miR-549a | 48.49 | 56.8 | 105.29 | 0.853697 |
| hsa-miR-370-3p | 48.49 | 54.61 | 103.1 | 0.887933 |
| hsa-miR-33a-5p | 48.49 | 53.52 | 102.01 | 0.906016 |
| hsa-miR-3182 | 48.49 | 52.43 | 100.92 | 0.924852 |
| hsa-miR-576-5p | 48.49 | 51.34 | 99.83 | 0.944488 |
| hsa-miR-584-5p | 48.49 | 45.88 | 94.37 | 1.056888 |
| hsa-miR-376c-3p | 48.49 | 44.78 | 93.27 | 1.082849 |
| hsa-miR-3613-5p | 48.49 | 43.69 | 92.18 | 1.109865 |
| hsa-miR-4536-3p | 47.56 | 78.65 | 126.21 | 0.604704 |
| hsa-miR-513a-3p | 47.56 | 78.65 | 126.21 | 0.604704 |
| hsa-miR-1287-5p | 47.56 | 74.28 | 121.84 | 0.64028 |
| hsa-miR-33b-5p | 47.56 | 67.72 | 115.28 | 0.702304 |
| hsa-miR-96-5p | 47.56 | 66.63 | 114.19 | 0.713793 |
| hsa-miR-556-5p | 47.56 | 63.35 | 110.91 | 0.75075 |
| hsa-miR-551a | 47.56 | 62.26 | 109.82 | 0.763893 |
| hsa-miR-410-3p | 47.56 | 61.17 | 108.73 | 0.777505 |
| hsa-miR-766-3p | 47.56 | 61.17 | 108.73 | 0.777505 |
| hsa-miR-423-3p | 47.56 | 60.08 | 107.64 | 0.791611 |
| hsa-miR-122-5p | 47.56 | 56.8 | 104.36 | 0.837324 |
| hsa-miR-510-3p | 47.56 | 55.71 | 103.27 | 0.853707 |
| hsa-miR-339-5p | 47.56 | 49.15 | 96.71 | 0.96765 |
| hsa-miR-369-5p | 47.56 | 49.15 | 96.71 | 0.96765 |
| hsa-miR-210-3p | 47.56 | 46.97 | 94.53 | 1.012561 |
| hsa-miR-509-5p | 47.56 | 46.97 | 94.53 | 1.012561 |
| hsa-miR-587 | 47.56 | 42.6 | 90.16 | 1.116432 |
| hsa-miR-219a-5p | 47.56 | 41.51 | 89.07 | 1.145748 |
| hsa-miR-99a-5p | 46.63 | 95.03 | 141.66 | 0.490687 |
| hsa-miR-942-5p | 46.63 | 79.74 | 126.37 | 0.584776 |
| hsa-miR-595 | 46.63 | 68.81 | 115.44 | 0.677663 |
| hsa-miR-1268a | 46.63 | 64.45 | 111.08 | 0.723507 |
| hsa-miR-181a-3p | 46.63 | 61.17 | 107.8 | 0.762302 |
| hsa-miR-506-5p | 46.63 | 58.98 | 105.61 | 0.790607 |
| hsa-miR-1252-5p | 46.63 | 46.97 | 93.6 | 0.992761 |
| hsa-miR-3613-3p | 45.69 | 83.01 | 128.7 | 0.550416 |
| hsa-miR-574-3p | 45.69 | 72.09 | 117.78 | 0.633791 |
| hsa-miR-1261 | 45.69 | 67.72 | 113.41 | 0.67469 |
| hsa-miR-181b-2-3p | 45.69 | 60.08 | 105.77 | 0.760486 |
| hsa-miR-1273c | 45.69 | 56.8 | 102.49 | 0.804401 |
| hsa-miR-484 | 45.69 | 56.8 | 102.49 | 0.804401 |
| hsa-miR-660-3p | 45.69 | 55.71 | 101.4 | 0.82014 |
| hsa-miR-1307-5p | 45.69 | 54.61 | 100.3 | 0.83666 |
| hsa-miR-614 | 45.69 | 54.61 | 100.3 | 0.83666 |
| hsa-miR-133a-5p | 45.69 | 50.25 | 95.94 | 0.909254 |
| hsa-miR-101-3p | 45.69 | 45.88 | 91.57 | 0.995859 |
| hsa-miR-30c-5p | 45.69 | 43.69 | 89.38 | 1.045777 |
| hsa-miR-532-5p | 45.69 | 42.6 | 88.29 | 1.072535 |
| hsa-miR-3127-5p | 45.69 | 40.42 | 86.11 | 1.130381 |
| hsa-miR-203a-5p | 44.76 | 76.46 | 121.22 | 0.585404 |
| hsa-miR-381-3p | 44.76 | 73.18 | 117.94 | 0.611643 |
| hsa-miR-485-3p | 44.76 | 67.72 | 112.48 | 0.660957 |
| hsa-miR-3151-5p | 44.76 | 61.17 | 105.93 | 0.731731 |
| hsa-miR-4787-3p | 44.76 | 56.8 | 101.56 | 0.788028 |
| hsa-miR-1254 | 44.76 | 55.71 | 100.47 | 0.803446 |
| hsa-miR-219b-3p | 44.76 | 50.25 | 95.01 | 0.890746 |
| hsa-miR-649 | 44.76 | 50.25 | 95.01 | 0.890746 |
| hsa-miR-1202 | 44.76 | 49.15 | 93.91 | 0.910682 |
| hsa-miR-937-3p | 44.76 | 49.15 | 93.91 | 0.910682 |
| hsa-miR-520d-3p | 44.76 | 48.06 | 92.82 | 0.931336 |
| hsa-miR-627-3p | 44.76 | 45.88 | 90.64 | 0.975588 |
| hsa-miR-208a-3p | 44.76 | 44.78 | 89.54 | 0.999553 |
| hsa-miR-3136-5p | 44.76 | 41.51 | 86.27 | 1.078294 |
| hsa-miR-1304-3p | 44.76 | 40.42 | 85.18 | 1.107373 |
| hsa-miR-483-3p | 44.76 | 34.95 | 79.71 | 1.280687 |
| hsa-miR-92a-3p | 44.76 | 34.95 | 79.71 | 1.280687 |
| hsa-miR-214-3p | 43.83 | 87.38 | 131.21 | 0.501602 |
| hsa-miR-802 | 43.83 | 75.37 | 119.2 | 0.581531 |
| hsa-miR-140-3p | 43.83 | 69.91 | 113.74 | 0.626949 |
| hsa-miR-548e-5p | 43.83 | 64.45 | 108.28 | 0.680062 |
| hsa-miR-767-5p | 43.83 | 63.35 | 107.18 | 0.691871 |
| hsa-miR-1909-3p | 43.83 | 60.08 | 103.91 | 0.729527 |
| hsa-miR-487b-5p | 43.83 | 53.52 | 97.35 | 0.818946 |
| hsa-miR-4647 | 43.83 | 52.43 | 96.26 | 0.835972 |
| hsa-miR-532-3p | 43.83 | 49.15 | 92.98 | 0.89176 |
| hsa-miR-563 | 43.83 | 48.06 | 91.89 | 0.911985 |
| hsa-miR-9-5p | 43.83 | 48.06 | 91.89 | 0.911985 |
| hsa-miR-1185-5p | 43.83 | 46.97 | 90.8 | 0.933149 |
| hsa-miR-5196-3p +  hsa-miR-6732-3p | 43.83 | 44.78 | 88.61 | 0.978785 |
| hsa-miR-499b-3p | 43.83 | 42.6 | 86.43 | 1.028873 |
| hsa-miR-548j-5p | 43.83 | 41.51 | 85.34 | 1.05589 |
| hsa-miR-652-3p | 43.83 | 41.51 | 85.34 | 1.05589 |
| hsa-miR-519b-5p +  hsa-miR-519c-5p +  hsa-miR-523-5p +  hsa-miR-518e-5p +  hsa-miR-522-5p +  hsa-miR-519a-5p | 43.83 | 37.14 | 80.97 | 1.180129 |
| hsa-miR-193a-3p | 43.83 | 34.95 | 78.78 | 1.254077 |
| hsa-miR-760 | 43.83 | 34.95 | 78.78 | 1.254077 |
| hsa-miR-650 | 42.9 | 69.91 | 112.81 | 0.613646 |
| hsa-miR-1272 | 42.9 | 60.08 | 102.98 | 0.714048 |
| hsa-miR-4707-5p | 42.9 | 60.08 | 102.98 | 0.714048 |
| hsa-miR-6503-5p | 42.9 | 58.98 | 101.88 | 0.727365 |
| hsa-miR-520h | 42.9 | 57.89 | 100.79 | 0.741061 |
| hsa-miR-485-5p | 42.9 | 55.71 | 98.61 | 0.770059 |
| hsa-miR-4792 | 42.9 | 54.61 | 97.51 | 0.78557 |
| hsa-miR-885-3p | 42.9 | 54.61 | 97.51 | 0.78557 |
| hsa-miR-30a-3p | 42.9 | 53.52 | 96.42 | 0.80157 |
| hsa-miR-1255a | 42.9 | 52.43 | 95.33 | 0.818234 |
| hsa-miR-339-3p | 42.9 | 52.43 | 95.33 | 0.818234 |
| hsa-miR-607 | 42.9 | 52.43 | 95.33 | 0.818234 |
| hsa-miR-455-5p | 42.9 | 51.34 | 94.24 | 0.835606 |
| hsa-miR-3131 | 42.9 | 48.06 | 90.96 | 0.892634 |
| hsa-miR-483-5p | 42.9 | 46.97 | 89.87 | 0.913349 |
| hsa-miR-638 | 42.9 | 44.78 | 87.68 | 0.958017 |
| hsa-miR-144-3p | 42.9 | 43.69 | 86.59 | 0.981918 |
| hsa-miR-552-3p | 42.9 | 36.05 | 78.95 | 1.190014 |
| hsa-miR-3195 | 42.9 | 32.77 | 75.67 | 1.309124 |
| hsa-miR-130b-3p | 42.9 | 30.58 | 73.48 | 1.402878 |
| hsa-miR-138-5p | 42.9 | 29.49 | 72.39 | 1.45473 |
| hsa-miR-553 | 42.9 | 29.49 | 72.39 | 1.45473 |
| hsa-miR-626 | 41.96 | 75.37 | 117.33 | 0.55672 |
| hsa-miR-4787-5p | 41.96 | 60.08 | 102.04 | 0.698402 |
| hsa-miR-34c-3p | 41.96 | 56.8 | 98.76 | 0.738732 |
| hsa-miR-376c-5p | 41.96 | 53.52 | 95.48 | 0.784006 |
| hsa-miR-570-3p | 41.96 | 46.97 | 88.93 | 0.893336 |
| hsa-miR-518c-3p | 41.96 | 42.6 | 84.56 | 0.984977 |
| hsa-miR-450a-2-3p | 41.96 | 41.51 | 83.47 | 1.010841 |
| hsa-miR-548y | 41.96 | 41.51 | 83.47 | 1.010841 |
| hsa-miR-513b-5p | 41.96 | 40.42 | 82.38 | 1.0381 |
| hsa-miR-500a-5p +  hsa-miR-501-5p | 41.96 | 21.85 | 63.81 | 1.920366 |
| hsa-miR-508-5p | 41.03 | 69.91 | 110.94 | 0.586897 |
| hsa-miR-224-5p | 41.03 | 62.26 | 103.29 | 0.659011 |
| hsa-miR-885-5p | 41.03 | 62.26 | 103.29 | 0.659011 |
| hsa-miR-187-3p | 41.03 | 61.17 | 102.2 | 0.670754 |
| hsa-miR-296-5p | 41.03 | 60.08 | 101.11 | 0.682923 |
| hsa-miR-3605-5p | 41.03 | 58.98 | 100.01 | 0.69566 |
| hsa-miR-382-5p | 41.03 | 57.89 | 98.92 | 0.708758 |
| hsa-miR-129-2-3p | 41.03 | 56.8 | 97.83 | 0.722359 |
| hsa-miR-4461 | 41.03 | 56.8 | 97.83 | 0.722359 |
| hsa-miR-1271-5p | 41.03 | 54.61 | 95.64 | 0.751328 |
| hsa-miR-515-5p | 41.03 | 54.61 | 95.64 | 0.751328 |
| hsa-miR-1976 | 41.03 | 52.43 | 93.46 | 0.782567 |
| hsa-miR-301a-5p | 41.03 | 51.34 | 92.37 | 0.799182 |
| hsa-miR-1260b | 41.03 | 49.15 | 90.18 | 0.834791 |
| hsa-miR-300 | 41.03 | 49.15 | 90.18 | 0.834791 |
| hsa-miR-129-5p | 41.03 | 48.06 | 89.09 | 0.853725 |
| hsa-miR-422a | 41.03 | 46.97 | 88 | 0.873536 |
| hsa-miR-302a-3p | 41.03 | 44.78 | 85.81 | 0.916257 |
| hsa-miR-320a | 41.03 | 44.78 | 85.81 | 0.916257 |
| hsa-miR-671-5p | 41.03 | 43.69 | 84.72 | 0.939117 |
| hsa-miR-661 | 41.03 | 40.42 | 81.45 | 1.015092 |
| hsa-miR-1299 | 41.03 | 39.32 | 80.35 | 1.043489 |
| hsa-miR-539-3p | 41.03 | 37.14 | 78.17 | 1.104739 |
| hsa-miR-770-5p | 41.03 | 37.14 | 78.17 | 1.104739 |
| hsa-miR-432-5p | 41.03 | 34.95 | 75.98 | 1.173963 |
| hsa-miR-514b-3p | 41.03 | 34.95 | 75.98 | 1.173963 |
| hsa-miR-32-5p | 41.03 | 16.38 | 57.41 | 2.504884 |
| hsa-miR-374c-5p | 40.1 | 61.17 | 101.27 | 0.65555 |
| hsa-miR-450a-5p | 40.1 | 58.98 | 99.08 | 0.679891 |
| hsa-miR-1236-3p | 40.1 | 57.89 | 97.99 | 0.692693 |
| hsa-miR-190a-3p | 40.1 | 56.8 | 96.9 | 0.705986 |
| hsa-miR-514a-3p | 40.1 | 56.8 | 96.9 | 0.705986 |
| hsa-miR-3168 | 40.1 | 53.52 | 93.62 | 0.749253 |
| hsa-miR-562 | 40.1 | 51.34 | 91.44 | 0.781067 |
| hsa-miR-448 | 40.1 | 50.25 | 90.35 | 0.79801 |
| hsa-miR-507 | 40.1 | 50.25 | 90.35 | 0.79801 |
| hsa-miR-412-3p | 40.1 | 48.06 | 88.16 | 0.834374 |
| hsa-miR-526a +  hsa-miR-518c-5p +  hsa-miR-518d-5p | 40.1 | 46.97 | 87.07 | 0.853736 |
| hsa-miR-1293 | 40.1 | 43.69 | 83.79 | 0.91783 |
| hsa-miR-433-3p | 40.1 | 43.69 | 83.79 | 0.91783 |
| hsa-miR-2053 | 40.1 | 42.6 | 82.7 | 0.941315 |
| hsa-miR-512-5p | 40.1 | 42.6 | 82.7 | 0.941315 |
| hsa-miR-3690 | 40.1 | 38.23 | 78.33 | 1.048914 |
| hsa-miR-190a-5p | 40.1 | 34.95 | 75.05 | 1.147353 |
| hsa-miR-4707-3p | 40.1 | 32.77 | 72.87 | 1.22368 |
| hsa-miR-325 | 39.17 | 65.54 | 104.71 | 0.59765 |
| hsa-miR-5001-5p | 39.17 | 63.35 | 102.52 | 0.618311 |
| hsa-miR-4425 | 39.17 | 58.98 | 98.15 | 0.664123 |
| hsa-miR-1234-3p | 39.17 | 57.89 | 97.06 | 0.676628 |
| hsa-miR-887-5p | 39.17 | 57.89 | 97.06 | 0.676628 |
| hsa-miR-498 | 39.17 | 55.71 | 94.88 | 0.703105 |
| hsa-miR-548ai +  hsa-miR-570-5p | 39.17 | 55.71 | 94.88 | 0.703105 |
| hsa-miR-566 | 39.17 | 51.34 | 90.51 | 0.762953 |
| hsa-miR-6720-3p | 39.17 | 50.25 | 89.42 | 0.779502 |
| hsa-miR-147b | 39.17 | 49.15 | 88.32 | 0.796948 |
| hsa-miR-610 | 39.17 | 46.97 | 86.14 | 0.833937 |
| hsa-miR-548l | 39.17 | 45.88 | 85.05 | 0.853749 |
| hsa-miR-518b | 39.17 | 43.69 | 82.86 | 0.896544 |
| hsa-miR-302b-3p | 39.17 | 41.51 | 80.68 | 0.943628 |
| hsa-miR-1205 | 39.17 | 40.42 | 79.59 | 0.969075 |
| hsa-miR-519d-3p | 39.17 | 40.42 | 79.59 | 0.969075 |
| hsa-miR-643 | 39.17 | 38.23 | 77.4 | 1.024588 |
| hsa-miR-874-3p | 39.17 | 37.14 | 76.31 | 1.054658 |
| hsa-miR-940 | 39.17 | 37.14 | 76.31 | 1.054658 |
| hsa-miR-128-2-5p | 39.17 | 32.77 | 71.94 | 1.195301 |
| hsa-miR-198 | 38.23 | 54.61 | 92.84 | 0.700055 |
| hsa-miR-3158-3p | 38.23 | 54.61 | 92.84 | 0.700055 |
| hsa-miR-1827 | 38.23 | 50.25 | 88.48 | 0.760796 |
| hsa-miR-195-5p | 38.23 | 48.06 | 86.29 | 0.795464 |
| hsa-miR-378b | 38.23 | 45.88 | 84.11 | 0.833261 |
| hsa-miR-503-3p | 38.23 | 45.88 | 84.11 | 0.833261 |
| hsa-miR-3614-3p | 38.23 | 44.78 | 83.01 | 0.853729 |
| hsa-miR-369-3p | 38.23 | 42.6 | 80.83 | 0.897418 |
| hsa-miR-518e-3p | 38.23 | 40.42 | 78.65 | 0.945819 |
| hsa-miR-302c-3p | 38.23 | 38.23 | 76.46 | 1 |
| hsa-miR-4435 | 38.23 | 38.23 | 76.46 | 1 |
| hsa-miR-1258 | 38.23 | 34.95 | 73.18 | 1.093848 |
| hsa-miR-323a-3p | 38.23 | 33.86 | 72.09 | 1.129061 |
| hsa-miR-367-3p | 38.23 | 33.86 | 72.09 | 1.129061 |
| hsa-miR-5010-5p | 38.23 | 33.86 | 72.09 | 1.129061 |
| hsa-miR-362-3p | 38.23 | 29.49 | 67.72 | 1.296372 |
| hsa-miR-487b-3p | 38.23 | 29.49 | 67.72 | 1.296372 |
| hsa-miR-505-3p | 38.23 | 28.4 | 66.63 | 1.346127 |
| hsa-miR-127-3p | 37.3 | 79.74 | 117.04 | 0.46777 |
| hsa-miR-1275 | 37.3 | 67.72 | 105.02 | 0.550797 |
| hsa-miR-1245a | 37.3 | 53.52 | 90.82 | 0.696936 |
| hsa-miR-1268b | 37.3 | 52.43 | 89.73 | 0.711425 |
| hsa-miR-372-3p | 37.3 | 49.15 | 86.45 | 0.758901 |
| hsa-miR-876-5p | 37.3 | 48.06 | 85.36 | 0.776113 |
| hsa-miR-491-5p | 37.3 | 46.97 | 84.27 | 0.794124 |
| hsa-miR-1278 | 37.3 | 44.78 | 82.08 | 0.832961 |
| hsa-miR-599 | 37.3 | 44.78 | 82.08 | 0.832961 |
| hsa-miR-874-5p | 37.3 | 44.78 | 82.08 | 0.832961 |
| hsa-miR-1178-3p | 37.3 | 43.69 | 80.99 | 0.853742 |
| hsa-miR-3065-5p | 37.3 | 43.69 | 80.99 | 0.853742 |
| hsa-miR-506-3p | 37.3 | 39.32 | 76.62 | 0.948627 |
| hsa-miR-766-5p | 37.3 | 32.77 | 70.07 | 1.138236 |
| hsa-miR-508-3p | 36.37 | 69.91 | 106.28 | 0.52024 |
| hsa-miR-582-3p | 36.37 | 58.98 | 95.35 | 0.61665 |
| hsa-miR-504-3p | 36.37 | 53.52 | 89.89 | 0.679559 |
| hsa-miR-542-5p | 36.37 | 53.52 | 89.89 | 0.679559 |
| hsa-miR-519c-3p | 36.37 | 48.06 | 84.43 | 0.756762 |
| hsa-miR-499b-5p | 36.37 | 44.78 | 81.15 | 0.812193 |
| hsa-miR-520g-3p | 36.37 | 44.78 | 81.15 | 0.812193 |
| hsa-miR-1185-2-3p | 36.37 | 43.69 | 80.06 | 0.832456 |
| hsa-miR-1302 | 36.37 | 43.69 | 80.06 | 0.832456 |
| hsa-miR-5196-5p | 36.37 | 43.69 | 80.06 | 0.832456 |
| hsa-miR-219a-2-3p | 36.37 | 42.6 | 78.97 | 0.853756 |
| hsa-miR-338-5p | 36.37 | 42.6 | 78.97 | 0.853756 |
| hsa-miR-520c-3p | 36.37 | 40.42 | 76.79 | 0.899802 |
| hsa-miR-576-3p | 36.37 | 40.42 | 76.79 | 0.899802 |
| hsa-miR-1-5p | 36.37 | 39.32 | 75.69 | 0.924975 |
| hsa-miR-1291 | 36.37 | 39.32 | 75.69 | 0.924975 |
| hsa-miR-512-3p | 36.37 | 39.32 | 75.69 | 0.924975 |
| hsa-miR-6511a-5p | 36.37 | 39.32 | 75.69 | 0.924975 |
| hsa-miR-184 | 36.37 | 38.23 | 74.6 | 0.951347 |
| hsa-miR-193b-3p | 36.37 | 38.23 | 74.6 | 0.951347 |
| hsa-miR-544a | 36.37 | 38.23 | 74.6 | 0.951347 |
| hsa-miR-504-5p | 36.37 | 36.05 | 72.42 | 1.008877 |
| hsa-miR-513c-3p | 36.37 | 36.05 | 72.42 | 1.008877 |
| hsa-miR-210-5p | 36.37 | 27.31 | 63.68 | 1.331747 |
| hsa-miR-654-3p | 36.37 | 26.22 | 62.59 | 1.387109 |
| hsa-miR-889-3p | 35.44 | 52.43 | 87.87 | 0.675949 |
| hsa-miR-892b | 35.44 | 51.34 | 86.78 | 0.6903 |
| hsa-miR-604 | 35.44 | 50.25 | 85.69 | 0.705274 |
| hsa-miR-3202 | 35.44 | 44.78 | 80.22 | 0.791425 |
| hsa-miR-520d-5p +  hsa-miR-527 +  hsa-miR-518a-5p | 35.44 | 44.78 | 80.22 | 0.791425 |
| hsa-miR-450b-3p | 35.44 | 42.6 | 78.04 | 0.831925 |
| hsa-miR-384 | 35.44 | 41.51 | 76.95 | 0.85377 |
| hsa-miR-554 | 35.44 | 40.42 | 75.86 | 0.876794 |
| hsa-miR-6503-3p | 35.44 | 39.32 | 74.76 | 0.901322 |
| hsa-miR-654-5p | 35.44 | 37.14 | 72.58 | 0.954227 |
| hsa-miR-1269b | 35.44 | 30.58 | 66.02 | 1.158927 |
| hsa-miR-520a-5p | 34.5 | 57.89 | 92.39 | 0.595958 |
| hsa-miR-516a-3p +  hsa-miR-516b-3p | 34.5 | 55.71 | 90.21 | 0.619278 |
| hsa-miR-1226-3p | 34.5 | 50.25 | 84.75 | 0.686567 |
| hsa-miR-486-3p | 34.5 | 46.97 | 81.47 | 0.734511 |
| hsa-miR-3065-3p | 34.5 | 44.78 | 79.28 | 0.770433 |
| hsa-miR-548ad-3p | 34.5 | 44.78 | 79.28 | 0.770433 |
| hsa-miR-551b-3p | 34.5 | 44.78 | 79.28 | 0.770433 |
| hsa-miR-665 | 34.5 | 43.69 | 78.19 | 0.789654 |
| hsa-miR-1266-5p | 34.5 | 42.6 | 77.1 | 0.809859 |
| hsa-miR-373-3p | 34.5 | 39.32 | 73.82 | 0.877416 |
| hsa-miR-605-5p | 34.5 | 39.32 | 73.82 | 0.877416 |
| hsa-miR-591 | 34.5 | 38.23 | 72.73 | 0.902433 |
| hsa-miR-517c-3p +  hsa-miR-519a-3p | 34.5 | 37.14 | 71.64 | 0.928918 |
| hsa-miR-548g-3p | 34.5 | 37.14 | 71.64 | 0.928918 |
| hsa-miR-452-5p | 34.5 | 36.05 | 70.55 | 0.957004 |
| hsa-miR-548j-3p | 34.5 | 36.05 | 70.55 | 0.957004 |
| hsa-miR-449a | 34.5 | 34.95 | 69.45 | 0.987124 |
| hsa-miR-769-3p | 34.5 | 34.95 | 69.45 | 0.987124 |
| hsa-miR-342-5p | 34.5 | 32.77 | 67.27 | 1.052792 |
| hsa-miR-596 | 34.5 | 30.58 | 65.08 | 1.128188 |
| hsa-miR-3180 | 34.5 | 29.49 | 63.99 | 1.169888 |
| hsa-miR-154-5p | 34.5 | 28.4 | 62.9 | 1.214789 |
| hsa-miR-133b | 34.5 | 26.22 | 60.72 | 1.315789 |
| hsa-miR-499a-3p | 33.57 | 54.61 | 88.18 | 0.614723 |
| hsa-miR-556-3p | 33.57 | 54.61 | 88.18 | 0.614723 |
| hsa-miR-1269a | 33.57 | 49.15 | 82.72 | 0.683011 |
| hsa-miR-125a-3p | 33.57 | 48.06 | 81.63 | 0.698502 |
| hsa-miR-1271-3p | 33.57 | 46.97 | 80.54 | 0.714712 |
| hsa-miR-193a-5p +  hsa-miR-193b-5p | 33.57 | 46.97 | 80.54 | 0.714712 |
| hsa-miR-376b-3p | 33.57 | 46.97 | 80.54 | 0.714712 |
| hsa-miR-1289 | 33.57 | 45.88 | 79.45 | 0.731691 |
| hsa-miR-573 | 33.57 | 43.69 | 77.26 | 0.768368 |
| hsa-miR-296-3p | 33.57 | 42.6 | 76.17 | 0.788028 |
| hsa-miR-584-3p | 33.57 | 41.51 | 75.08 | 0.808721 |
| hsa-miR-631 | 33.57 | 41.51 | 75.08 | 0.808721 |
| hsa-miR-548o-3p +  hsa-miR-548ah-3p +  hsa-miR-548av-3p | 33.57 | 40.42 | 73.99 | 0.830529 |
| hsa-miR-671-3p | 33.57 | 38.23 | 71.8 | 0.878106 |
| hsa-miR-6724-5p | 33.57 | 32.77 | 66.34 | 1.024413 |
| hsa-miR-1264 | 33.57 | 30.58 | 64.15 | 1.097776 |
| hsa-miR-617 | 33.57 | 27.31 | 60.88 | 1.22922 |
| hsa-miR-548a-3p | 33.57 | 25.12 | 58.69 | 1.336385 |
| hsa-miR-363-5p | 33.57 | 19.66 | 53.23 | 1.707528 |
| hsa-miR-1262 | 32.64 | 53.52 | 86.16 | 0.609865 |
| hsa-miR-345-5p | 32.64 | 50.25 | 82.89 | 0.649552 |
| hsa-miR-520b | 32.64 | 48.06 | 80.7 | 0.679151 |
| hsa-miR-758-3p +  hsa-miR-411-3p | 32.64 | 48.06 | 80.7 | 0.679151 |
| hsa-miR-34c-5p | 32.64 | 44.78 | 77.42 | 0.728897 |
| hsa-miR-513a-5p | 32.64 | 41.51 | 74.15 | 0.786317 |
| hsa-miR-517b-3p | 32.64 | 41.51 | 74.15 | 0.786317 |
| hsa-miR-190b | 32.64 | 36.05 | 68.69 | 0.905409 |
| hsa-miR-590-3p | 32.64 | 36.05 | 68.69 | 0.905409 |
| hsa-miR-1-3p | 32.64 | 33.86 | 66.5 | 0.963969 |
| hsa-miR-323b-5p | 32.64 | 32.77 | 65.41 | 0.996033 |
| hsa-miR-936 | 32.64 | 32.77 | 65.41 | 0.996033 |
| hsa-miR-548e-3p | 32.64 | 31.68 | 64.32 | 1.030303 |
| hsa-miR-664b-5p | 32.64 | 27.31 | 59.95 | 1.195167 |
| hsa-miR-1185-1-3p | 31.71 | 69.91 | 101.62 | 0.453583 |
| hsa-miR-1306-5p | 31.71 | 50.25 | 81.96 | 0.631045 |
| hsa-miR-328-5p | 31.71 | 50.25 | 81.96 | 0.631045 |
| hsa-miR-521 | 31.71 | 50.25 | 81.96 | 0.631045 |
| hsa-miR-675-5p | 31.71 | 50.25 | 81.96 | 0.631045 |
| hsa-miR-1203 | 31.71 | 49.15 | 80.86 | 0.645168 |
| hsa-miR-208b-3p | 31.71 | 46.97 | 78.68 | 0.675112 |
| hsa-miR-616-3p | 31.71 | 44.78 | 76.49 | 0.708129 |
| hsa-miR-330-5p | 31.71 | 42.6 | 74.31 | 0.744366 |
| hsa-miR-571 | 31.71 | 41.51 | 73.22 | 0.763912 |
| hsa-miR-331-5p | 31.71 | 36.05 | 67.76 | 0.879612 |
| hsa-miR-520a-3p | 31.71 | 30.58 | 62.29 | 1.036952 |
| hsa-miR-490-3p | 31.71 | 28.4 | 60.11 | 1.116549 |
| hsa-miR-941 | 31.71 | 28.4 | 60.11 | 1.116549 |
| hsa-miR-383-5p | 31.71 | 20.75 | 52.46 | 1.528193 |
| hsa-miR-329-5p | 30.77 | 49.15 | 79.92 | 0.626043 |
| hsa-miR-1193 | 30.77 | 46.97 | 77.74 | 0.655099 |
| hsa-miR-134-3p | 30.77 | 44.78 | 75.55 | 0.687137 |
| hsa-miR-548h-5p | 30.77 | 40.42 | 71.19 | 0.761257 |
| hsa-miR-124-3p | 30.77 | 37.14 | 67.91 | 0.828487 |
| hsa-miR-655-3p | 30.77 | 36.05 | 66.82 | 0.853537 |
| hsa-miR-409-5p | 30.77 | 34.95 | 65.72 | 0.880401 |
| hsa-miR-1910-3p | 30.77 | 33.86 | 64.63 | 0.908742 |
| hsa-miR-1250-5p | 30.77 | 32.77 | 63.54 | 0.938969 |
| hsa-miR-944 | 30.77 | 32.77 | 63.54 | 0.938969 |
| hsa-miR-548ak | 30.77 | 31.68 | 62.45 | 0.971275 |
| hsa-miR-518d-3p | 30.77 | 30.58 | 61.35 | 1.006213 |
| hsa-miR-3916 | 30.77 | 29.49 | 60.26 | 1.043405 |
| hsa-miR-302a-5p | 30.77 | 25.12 | 55.89 | 1.22492 |
| hsa-miR-628-3p | 29.84 | 53.52 | 83.36 | 0.557549 |
| hsa-miR-3615 | 29.84 | 52.43 | 82.27 | 0.56914 |
| hsa-miR-620 | 29.84 | 46.97 | 76.81 | 0.635299 |
| hsa-miR-541-3p | 29.84 | 44.78 | 74.62 | 0.666369 |
| hsa-miR-2110 | 29.84 | 43.69 | 73.53 | 0.682994 |
| hsa-miR-105-5p | 29.84 | 39.32 | 69.16 | 0.758901 |
| hsa-miR-758-5p | 29.84 | 37.14 | 66.98 | 0.803446 |
| hsa-miR-1287-3p | 29.84 | 34.95 | 64.79 | 0.853791 |
| hsa-miR-513c-5p | 29.84 | 33.86 | 63.7 | 0.881276 |
| hsa-miR-1288-3p | 29.84 | 29.49 | 59.33 | 1.011868 |
| hsa-miR-3934-5p | 29.84 | 29.49 | 59.33 | 1.011868 |
| hsa-miR-3605-3p | 29.84 | 28.4 | 58.24 | 1.050704 |
| hsa-miR-516a-5p | 29.84 | 27.31 | 57.15 | 1.09264 |
| hsa-miR-328-3p | 29.84 | 22.94 | 52.78 | 1.300785 |
| hsa-miR-518f-3p | 29.84 | 21.85 | 51.69 | 1.365675 |
| hsa-miR-378d | 28.91 | 58.98 | 87.89 | 0.490166 |
| hsa-miR-1301-3p | 28.91 | 41.51 | 70.42 | 0.696459 |
| hsa-miR-548c-5p +  hsa-miR-548o-5p +  hsa-miR-548am-5p | 28.91 | 40.42 | 69.33 | 0.71524 |
| hsa-miR-376a-2-5p | 28.91 | 39.32 | 68.23 | 0.735249 |
| hsa-miR-433-5p | 28.91 | 38.23 | 67.14 | 0.756212 |
| hsa-miR-103a-3p | 28.91 | 37.14 | 66.05 | 0.778406 |
| hsa-miR-4458 | 28.91 | 37.14 | 66.05 | 0.778406 |
| hsa-miR-1244 | 28.91 | 34.95 | 63.86 | 0.827182 |
| hsa-miR-873-5p | 28.91 | 31.68 | 60.59 | 0.912563 |
| hsa-miR-887-3p | 28.91 | 31.68 | 60.59 | 0.912563 |
| hsa-miR-1204 | 28.91 | 30.58 | 59.49 | 0.945389 |
| hsa-miR-545-3p | 28.91 | 29.49 | 58.4 | 0.980332 |
| hsa-miR-2116-5p | 28.91 | 28.4 | 57.31 | 1.017958 |
| hsa-miR-3192-5p | 28.91 | 28.4 | 57.31 | 1.017958 |
| hsa-miR-1248 | 28.91 | 27.31 | 56.22 | 1.058587 |
| hsa-miR-492 | 28.91 | 26.22 | 55.13 | 1.102593 |
| hsa-miR-181a-2-3p | 27.98 | 76.46 | 104.44 | 0.365943 |
| hsa-miR-3928-3p | 27.98 | 51.34 | 79.32 | 0.544994 |
| hsa-miR-555 | 27.98 | 50.25 | 78.23 | 0.556816 |
| hsa-miR-320b | 27.98 | 39.32 | 67.3 | 0.711597 |
| hsa-miR-98-3p | 27.98 | 39.32 | 67.3 | 0.711597 |
| hsa-miR-708-5p | 27.98 | 36.05 | 64.03 | 0.776144 |
| hsa-miR-490-5p | 27.98 | 34.95 | 62.93 | 0.800572 |
| hsa-miR-92b-3p | 27.98 | 33.86 | 61.84 | 0.826344 |
| hsa-miR-3190-3p | 27.98 | 31.68 | 59.66 | 0.883207 |
| hsa-miR-330-3p | 27.98 | 31.68 | 59.66 | 0.883207 |
| hsa-miR-3918 | 27.98 | 30.58 | 58.56 | 0.914977 |
| hsa-miR-652-5p | 27.98 | 28.4 | 56.38 | 0.985211 |
| hsa-miR-1296-5p | 27.98 | 27.31 | 55.29 | 1.024533 |
| hsa-miR-567 | 27.98 | 18.57 | 46.55 | 1.506731 |
| hsa-miR-153-3p | 27.04 | 51.34 | 78.38 | 0.526685 |
| hsa-miR-561-3p | 27.04 | 44.78 | 71.82 | 0.603841 |
| hsa-miR-450b-5p | 27.04 | 38.23 | 65.27 | 0.707298 |
| hsa-miR-139-5p | 27.04 | 37.14 | 64.18 | 0.728056 |
| hsa-miR-297 | 27.04 | 36.05 | 63.09 | 0.750069 |
| hsa-miR-378c | 27.04 | 34.95 | 61.99 | 0.773677 |
| hsa-miR-1245b-3p | 27.04 | 31.68 | 58.72 | 0.853535 |
| hsa-miR-3164 | 27.04 | 30.58 | 57.62 | 0.884238 |
| hsa-miR-578 | 27.04 | 27.31 | 54.35 | 0.990114 |
| hsa-miR-4521 | 27.04 | 20.75 | 47.79 | 1.303133 |
| hsa-miR-450a-1-3p | 27.04 | 18.57 | 45.61 | 1.456112 |
| hsa-miR-5001-3p | 26.11 | 52.43 | 78.54 | 0.497997 |
| hsa-miR-561-5p | 26.11 | 41.51 | 67.62 | 0.629005 |
| hsa-miR-491-3p | 26.11 | 39.32 | 65.43 | 0.664039 |
| hsa-miR-1249-5p | 26.11 | 37.14 | 63.25 | 0.703016 |
| hsa-miR-1908-5p | 26.11 | 37.14 | 63.25 | 0.703016 |
| hsa-miR-449b-5p | 26.11 | 34.95 | 61.06 | 0.747067 |
| hsa-miR-637 | 26.11 | 32.77 | 58.88 | 0.796765 |
| hsa-miR-501-3p | 26.11 | 31.68 | 57.79 | 0.824179 |
| hsa-miR-4524a-5p | 26.11 | 28.4 | 54.51 | 0.919366 |
| hsa-miR-1298-5p | 26.11 | 25.12 | 51.23 | 1.039411 |
| hsa-miR-217 | 25.18 | 38.23 | 63.41 | 0.658645 |
| hsa-miR-651-3p | 25.18 | 38.23 | 63.41 | 0.658645 |
| hsa-miR-3130-3p | 25.18 | 37.14 | 62.32 | 0.677975 |
| hsa-miR-564 | 25.18 | 30.58 | 55.76 | 0.823414 |
| hsa-miR-601 | 25.18 | 30.58 | 55.76 | 0.823414 |
| hsa-miR-519e-3p | 25.18 | 29.49 | 54.67 | 0.853849 |
| hsa-miR-579-5p | 25.18 | 26.22 | 51.4 | 0.960336 |
| hsa-miR-211-3p | 25.18 | 19.66 | 44.84 | 1.280773 |
| hsa-miR-641 | 24.25 | 44.78 | 69.03 | 0.541536 |
| hsa-miR-380-3p | 24.25 | 42.6 | 66.85 | 0.569249 |
| hsa-miR-767-3p | 24.25 | 41.51 | 65.76 | 0.584197 |
| hsa-miR-18b-5p | 24.25 | 37.14 | 61.39 | 0.652935 |
| hsa-miR-924 | 24.25 | 37.14 | 61.39 | 0.652935 |
| hsa-miR-1908-3p | 24.25 | 33.86 | 58.11 | 0.716184 |
| hsa-miR-1910-5p | 24.25 | 33.86 | 58.11 | 0.716184 |
| hsa-miR-3179 | 24.25 | 33.86 | 58.11 | 0.716184 |
| hsa-miR-520e | 24.25 | 33.86 | 58.11 | 0.716184 |
| hsa-miR-2278 | 24.25 | 31.68 | 55.93 | 0.765467 |
| hsa-miR-606 | 24.25 | 30.58 | 54.83 | 0.793002 |
| hsa-miR-320c | 24.25 | 27.31 | 51.56 | 0.887953 |
| hsa-miR-449c-5p | 24.25 | 27.31 | 51.56 | 0.887953 |
| hsa-miR-204-5p | 24.25 | 26.22 | 50.47 | 0.924867 |
| hsa-miR-221-5p | 24.25 | 26.22 | 50.47 | 0.924867 |
| hsa-miR-31-5p | 24.25 | 26.22 | 50.47 | 0.924867 |
| hsa-miR-6511a-3p | 24.25 | 25.12 | 49.37 | 0.965366 |
| hsa-miR-935 | 24.25 | 25.12 | 49.37 | 0.965366 |
| hsa-miR-365b-5p | 24.25 | 22.94 | 47.19 | 1.057105 |
| hsa-miR-329-3p | 24.25 | 20.75 | 45 | 1.168675 |
| hsa-miR-519b-3p | 24.25 | 17.48 | 41.73 | 1.3873 |
| hsa-miR-3144-5p | 23.31 | 42.6 | 65.91 | 0.547183 |
| hsa-miR-146b-3p | 23.31 | 40.42 | 63.73 | 0.576695 |
| hsa-miR-3140-5p | 23.31 | 40.42 | 63.73 | 0.576695 |
| hsa-miR-615-5p | 23.31 | 38.23 | 61.54 | 0.609731 |
| hsa-miR-3196 | 23.31 | 37.14 | 60.45 | 0.627625 |
| hsa-miR-409-3p | 23.31 | 36.05 | 59.36 | 0.646602 |
| hsa-miR-548b-3p | 23.31 | 34.95 | 58.26 | 0.666953 |
| hsa-miR-1224-5p | 23.31 | 31.68 | 54.99 | 0.735795 |
| hsa-miR-323a-5p | 23.31 | 30.58 | 53.89 | 0.762263 |
| hsa-miR-3180-5p | 23.31 | 29.49 | 52.8 | 0.790437 |
| hsa-miR-510-5p | 23.31 | 29.49 | 52.8 | 0.790437 |
| hsa-miR-1285-3p | 23.31 | 27.31 | 50.62 | 0.853534 |
| hsa-miR-514a-5p | 23.31 | 27.31 | 50.62 | 0.853534 |
| hsa-miR-524-3p | 23.31 | 25.12 | 48.43 | 0.927946 |
| hsa-miR-568 | 23.31 | 19.66 | 42.97 | 1.185656 |
| hsa-miR-628-5p | 22.38 | 46.97 | 69.35 | 0.476474 |
| hsa-miR-619-3p | 22.38 | 43.69 | 66.07 | 0.512245 |
| hsa-miR-494-5p | 22.38 | 42.6 | 64.98 | 0.525352 |
| hsa-miR-202-3p | 22.38 | 20.75 | 43.13 | 1.078554 |
| hsa-miR-520f-3p | 22.38 | 19.66 | 42.04 | 1.138352 |
| hsa-miR-1247-5p | 21.45 | 32.77 | 54.22 | 0.654562 |
| hsa-miR-3150b-3p | 21.45 | 29.49 | 50.94 | 0.727365 |
| hsa-miR-488-3p | 21.45 | 28.4 | 49.85 | 0.755282 |
| hsa-miR-934 | 21.45 | 24.03 | 45.48 | 0.892634 |
| hsa-miR-139-3p | 20.52 | 45.88 | 66.4 | 0.447254 |
| hsa-miR-613 | 20.52 | 34.95 | 55.47 | 0.587124 |
| hsa-miR-208b-5p | 20.52 | 22.94 | 43.46 | 0.894507 |
| hsa-miR-151b | 20.52 | 20.75 | 41.27 | 0.988916 |
| hsa-miR-642a-5p | 19.58 | 44.78 | 64.36 | 0.437249 |
| hsa-miR-374a-3p | 19.58 | 34.95 | 54.53 | 0.560229 |
| hsa-miR-152-5p | 19.58 | 31.68 | 51.26 | 0.618056 |
| hsa-miR-580-3p | 19.58 | 25.12 | 44.7 | 0.779459 |
| hsa-miR-34b-3p | 19.58 | 20.75 | 40.33 | 0.943614 |
| hsa-miR-4421 | 19.58 | 19.66 | 39.24 | 0.995931 |
| hsa-miR-942-3p | 19.58 | 18.57 | 38.15 | 1.054389 |
| hsa-miR-892a | 19.58 | 14.2 | 33.78 | 1.378873 |
| hsa-miR-1233-3p | 18.65 | 43.69 | 62.34 | 0.426871 |
| hsa-miR-522-3p | 18.65 | 37.14 | 55.79 | 0.502154 |
| hsa-miR-875-3p | 18.65 | 30.58 | 49.23 | 0.609876 |
| hsa-miR-370-5p | 17.72 | 37.14 | 54.86 | 0.477114 |
| hsa-miR-320d | 17.72 | 28.4 | 46.12 | 0.623944 |
| hsa-miR-515-3p | 17.72 | 27.31 | 45.03 | 0.648847 |
| hsa-miR-323b-3p | 17.72 | 24.03 | 41.75 | 0.737412 |
| hsa-miR-142-5p | 17.72 | 21.85 | 39.57 | 0.810984 |
| hsa-miR-431-5p | 16.79 | 20.75 | 37.54 | 0.809157 |
| hsa-miR-516b-5p | 16.79 | 20.75 | 37.54 | 0.809157 |
| hsa-miR-382-3p | 15.85 | 41.51 | 57.36 | 0.381836 |
| hsa-miR-381-5p | 15.85 | 27.31 | 43.16 | 0.580373 |
| hsa-miR-523-3p | 15.85 | 22.94 | 38.79 | 0.690933 |
| hsa-miR-3074-3p | 15.85 | 17.48 | 33.33 | 0.906751 |
| hsa-miR-525-3p | 14.92 | 20.75 | 35.67 | 0.719036 |
| hsa-miR-550a-5p | 13.06 | 27.31 | 40.37 | 0.478213 |
| hsa-miR-326 | 13.06 | 12.02 | 25.08 | 1.086522 |
| hsa-miR-3140-3p | 12.12 | 14.2 | 26.32 | 0.853521 |
| hsa-miR-509-3p | 9.33 | 4.37 | 13.7 | 2.135011 |
