# Supplementary material for: Spatial expression analyses of the putative oncogene ciRS-7 in cancer reshape the microRNA sponge theory
Source: Nat Commun. 2020 Sep 11;11:4551. doi: 10.1038/s41467-020-18355-2 (PMC7486402; doi:10.1038/s41467-020-18355-2)
Supplement: Supplementary file 5 — Reporting Summary [file 41467_2020_18355_MOESM5_ESM.pdf]

nature research

Corresponding author(s): Dr. Lasse Sommer Kristensen and Dr. Henrik Hager

Last updated by author(s): Aug 5, 2020

# Reporting Summary

Nature Research wishes to improve the reproducibility of the work that we publish. This form provides structure for consistency and transparency in reporting. For further information on Nature Research policies, see Authors & Referees and the Editorial Policy Checklist.

## Statistics

For all statistical analyses, confirm that the following items are present in the figure legend, table legend, main text, or Methods section.

| n/a | Confirmed | |
|---|---|---|
| ☐ | ☒ | The exact sample size (*n*) for each experimental group/condition, given as a discrete number and unit of measurement |
| ☐ | ☒ | A statement on whether measurements were taken from distinct samples or whether the same sample was measured repeatedly |
| ☐ | ☒ | The statistical test(s) used AND whether they are one- or two-sided<br>*Only common tests should be described solely by name; describe more complex techniques in the Methods section.* |
| ☐ | ☒ | A description of all covariates tested |
| ☐ | ☒ | A description of any assumptions or corrections, such as tests of normality and adjustment for multiple comparisons |
| ☐ | ☒ | A full description of the statistical parameters including central tendency (e.g. means) or other basic estimates (e.g. regression coefficient) AND variation (e.g. standard deviation) or associated estimates of uncertainty (e.g. confidence intervals) |
| ☐ | ☒ | For null hypothesis testing, the test statistic (e.g. *F*, *t*, *r*) with confidence intervals, effect sizes, degrees of freedom and *P* value noted<br>*Give P values as exact values whenever suitable.* |
| ☒ | ☐ | For Bayesian analysis, information on the choice of priors and Markov chain Monte Carlo settings |
| ☒ | ☐ | For hierarchical and complex designs, identification of the appropriate level for tests and full reporting of outcomes |
| ☐ | ☒ | Estimates of effect sizes (e.g. Cohen's *d*, Pearson's *r*), indicating how they were calculated |

*Our web collection on statistics for biologists contains articles on many of the points above.*

## Software and code

Policy information about availability of computer code

| | |
|---|---|
| Data collection | Excel 2016 spreadsheet.<br>Hamamatsu NanoZoomer XR digital slide scanner. |
| Data analysis | The following software was used for data analysis:<br>NanoString nCounter data were analyzed using nSOLVER 4.0 software (NanoString Technologies), which is freely available at https://www.nanostring.com/products/analysis-software/nsolver.<br>LightCycler 480 SW 1.5.1 software.<br>Graphpad Prism version 7 software.<br>ImageJ software version 2.0.0-rc-69/1.52p.<br>NDPview 2 software version 2.7.52 for mac<br>R software version 3.5.1.<br><br>No costum code was developed in this study.<br>R code was from STHDA, Statistical tools for high-throughput data analysis (http://www.sthda.com/). |

For manuscripts utilizing custom algorithms or software that are central to the research but not yet described in published literature, software must be made available to editors/reviewers. We strongly encourage code deposition in a community repository (e.g. GitHub). See the Nature Research guidelines for submitting code & software for further information.

## Data

Policy information about availability of data

All manuscripts must include a data availability statement. This statement should provide the following information, where applicable:

- Accession codes, unique identifiers, or web links for publicly available datasets
- A list of figures that have associated raw data
- A description of any restrictions on data availability

The source data underlying Figs. 1c-h, m, 2a-f, 4 and 5 and Supplementary Figs. 5 and 7 are provided as a Source Data file. All other original data that support the findings of this study are available from the corresponding author upon reasonable request.

# Field-specific reporting

Please select the one below that is the best fit for your research. If you are not sure, read the appropriate sections before making your selection.

☒ Life sciences ☐ Behavioural & social sciences ☐ Ecological, evolutionary & environmental sciences

For a reference copy of the document with all sections, see nature.com/documents/nr-reporting-summary-flat.pdf

# Life sciences study design

All studies must disclose on these points even when the disclosure is negative.

| | |
|---|---|
| Sample size | No methods were used to predetermine sample size for the experiments, as this study originally was designed as a pilot study. However, because ciRS-7 turned out to be completely absent in the cancer cells in all patient samples and because most of the observed correlations with miR-7 target genes were highly significant, the sample size was deemed sufficient. |
| Data exclusions | One sample was excluded due to a partially failed lane in the NanoString nCounter experiment that resulted in an overall lack of data and gave rise to a quality control flag (mRNA Content Normalization Flag) in the data analyses using the nSOLVER 4.0 software. |
| Replication | No replication was done on individual patient samples given limited tissue availability. However, the main findings were shown using two completely independent methodologies and corroborated by additional experiments, which were performed in technical duplicate or triplicate. All attempts at replication was successful. |
| Randomization | Randomization was not relevant in this study as no samples were allocated into different experimental groups. |
| Blinding | Because there was no allocation of patients or animals into different experimental groups blinding of the investigators to groups during data collection and analyses was not relevant. However, the chromogenic in situ hybridization (CISH) analyses for ciRS-7 and the NanoString nCounter analyses were performed by two independent researchers in a blinded fashion. |

# Reporting for specific materials, systems and methods

We require information from authors about some types of materials, experimental systems and methods used in many studies. Here, indicate whether each material, system or method listed is relevant to your study. If you are not sure if a list item applies to your research, read the appropriate section before selecting a response.

### Materials & experimental systems

| n/a | Involved in the study | |
|---|---|---|
| ☐ | ☒ | Antibodies |
| ☐ | ☒ | Eukaryotic cell lines |
| ☒ | ☐ | Palaeontology |
| ☒ | ☐ | Animals and other organisms |
| ☐ | ☒ | Human research participants |
| ☒ | ☐ | Clinical data |

### Methods

| n/a | Involved in the study | |
|---|---|---|
| ☒ | ☐ | ChIP-seq |
| ☒ | ☐ | Flow cytometry |
| ☒ | ☐ | MRI-based neuroimaging |

## Antibodies

| | |
|---|---|
| Antibodies used | c-FOS (E8): sc-166940 (Santa Cruz Biotechnology, Dallas, Texas, USA) |
| Validation | The antibody is specific for an epitope mapping between amino acids 128-152 within an internal region of FOS (c-FOS) of human origin and is recommended for detection of c-FOS in human tissue using immunohistochemistry on FFPE tissues. Specificity of the antibody was tested by the manufacturer using Western blot analysis of FOS expression in non-transfected (sc-117752) and human FOS transfected (sc-112170 293T) whole cell lysates according to the accompanying data-sheet. We determined the optimal pretreatment conditions, antibody concentration and incubation time as well as evaluated the specificity of the antibody |

using human term placenta. A clear nuclear staining of the decidual cells and an absent background staining was achieved using the staining characteristics described in the Method section. We used isotope specific antibody as negative control.

## Eukaryotic cell lines

Policy information about cell lines

| | |
|---|---|
| Cell line source(s) | HEK293T cells purchased from ATCC. |
| Authentication | The cell line was not authenticated. |
| Mycoplasma contamination | The cell line has been tested negative for mycoplasma. |
| Commonly misidentified lines (See ICLAC register) | No commonly misidentified cell lines were used in this study. |

## Human research participants

Policy information about studies involving human research participants

Population characteristics

The patient population had the following characteristics:

Age (years)
<65, n (%) 6 (18.8)
≥65, n (%) 26 (81.2)

Sex
Male, n (%) 17 (53.1)
Female, n (%) 15 (46.9)

Primary tumor location
Cecum, n (%) 7 (21.9)
Colon ascendens, n (%) 3 (9.4)
Right Colic Flexure, n (%) 2 (6.3)
Colon Transversum, n (%) 6 (18.9)
Left Colic Flexure, n (%) 0 (0.0)
Colon descendens, n (%) 0 (0.0)
Colon sigmoideum, n (%) 14 (43.8)

Perineural invasion
Yes, n (%) 2 (6.3)
No, n (%) 21 (65.6)
Unknown, n (%) 9 (28.1)

Lymphatic or vascular invasion
Yes, n (%) 5 (15.6)
No, n (%) 22 (68.8)
Unknown, n (%) 5 (15.6)

Tumor differentiation
Well or moderately differentiated, n (%) 27 (84.4)
Poorly differentiated or undifferentiated, n (%) 5 (15.6)

Mismatch repair status
Mismatch repair-deficient, n (%) 6 (18.8)
Mismatch repair-proficient, n (%) 26 (81.3)
Unexamined, n (%) 0 (0.0)

T stage
T1, n (%) 0 (0.0)
T2, n (%) 0 (0.0)
T3, n (%) 27 (84.4)
T4, n (%) 5 (15.6)

N stage
N0, n (%) 27 (84.4)
N1, n (%) 5 (15.6)

M stage
M0, n (%) 32 (100.0)
M1, n (%) 0 (0.0)

Recruitment

This retrospective study was based on excess surgical specimens from patients with colon cancer collected at the Department of

| | |
|---|---|
| Recruitment | Clinical Pathology, Vejle Hospital, Denmark. All patients were treated surgically for stage II or III colon cancer in 2002 in Denmark and identified by a search in the nationwide registry administered by the Danish Colorectal Cancer Group (DCCG). We initially included 27 stage II patients randomly. Subsequently, we included 5 stage III patients randomly. Therefore, a bias towards more stage II than stage III patients are present in the study. This bias is unlikely to have impacted the results as the cancer cells were negative for ciRS-7 expression in 100% of the samples tested. All patients were screened in the Danish Registry of Tissue Utilization before enrollment in the study. |
| Ethics oversight | This study, including the use of patient samples, has been approved by the Regional Ethical Committee of Southern Denmark according to the Danish law (approval number: S-20170197 CSF). |

Note that full information on the approval of the study protocol must also be provided in the manuscript.
